# Impact of Multiple HVAC Systems on Indoor Air VOC and Radon Concentrations from Vapor Intrusion During Seasonal Usage

**DOI:** 10.3390/atmos16040378

**Published:** 2025-03-27

**Authors:** John H. Zimmerman, Alan Williams, Brian Schumacher, Christopher Lutes, Rohit Warrier, Brian Cosky, Ben Thompson, Chase W. Holton, Kate Bronstein

**Affiliations:** 1 Office of Research and Development, U.S. Environmental Protection Agency, 109 TW Alexander Dr., P.O. Box 12055, Research Triangle Park, Durham, NC 27711, USA; 2 Office of Research and Development, U.S. Environmental Protection Agency, 960 College Station Road, Athens, GA 30605, USA; 3 Jacobs, 1999 Bryan Street, Suite 1200, Dallas, TX 75201, USA; 4 GSI Environmental Inc., 13949 W. Colfax, No. 210, Lakewood, CO 80401, USA; 5 RTI International, 3040 E. Cornwallis Rd., Research Triangle Park, Durham, NC 27709, USA

**Keywords:** reasonable maximum exposure, indicators and tracers, subslab soil gas, vapor intrusion, passive sampling, volatile organic compounds

## Abstract

Subsurface contamination can migrate upward into overlying buildings, exposing the buildings’ inhabitants to contaminants that can cause detrimental health effects. This phenomenon is known as vapor intrusion (VI). When evaluating a building for VI, one must understand that seasonal and short-term variability are significant factors in determining the reasonable maximum exposure (RME) to the occupants. RME is a semi-quantitative term that refers to the lower portion of the high end of the exposure distribution—conceptually, above the 90th percentile exposure but less than the 98th percentile exposure. Samples were collected between December 2020 and April 2022 at six non-residential commercial buildings in Fairbanks, Alaska. The types of samples collected included indoor air (IA); outdoor air; subslab soil gas; soil gas; indoor radon; differential pressure; indoor and outdoor temperature; heating, ventilation, and air conditioning (HVAC) parameters; and other environmental factors. The buildings in close proximity to the volatile organic compound (VOC) source/release points presented less variability in indoor air concentrations of trichloroethylene (TCE) and tetrachloroethylene (PCE) compared to the buildings farther down gradient in the contaminated groundwater plume. The VOC data pattern for the source area buildings shows an outdoor air temperature-dominated behavior for indoor air concentrations in the summer season. HVAC system operations had less influence on long-term indoor air concentration trends than environmental factors, which is supported by similar indoor air concentration patterns independent of location within the plume. The use of soil temperature and indoor/outdoor temperatures as indicators and tracers (I&Ts) across the plume as predictors of the sampling period could produce a good estimation of the RME for the building occupants. These results, which show the use of soil temperature and indoor/outdoor temperatures as I&Ts, will help advance investigative methods for evaluation of VI in similar settings and thereby improve the protection of human health in indoor environments.

## 1. Introduction

This paper examines the seasonal and short-term temporal variability of volatile organic compounds (VOCs) measured in indoor air and subslab soil gas (SSSG) samples collected at six commercial buildings in Fairbanks, Alaska (Table 1 and Figure S1). Passive and active volatile organic compound (VOC) measurements were collected weekly for 15 months between December 2020 and April 2022. Various indicators and tracers (I&Ts), including indoor radon, differential pressure, temperature, and other environmental factors, were also collected. The results from this study help clarify the factors that influence temporal trends in VOC concentrations across multiple sampling zones in commercial buildings in a subarctic climate zone and thus inform efficient vapor intrusion sampling strategies.

### Background

Vapor intrusion (VI) is the migration of hazardous vapors from a subsurface contaminant source into an overlying building or structure via any route, opening, or conduit, posing health risks to occupants. VI is a complex process influenced by a variety of geological, meteorological, and building factors that can cause substantial temporal variability in indoor air VOC concentrations [1-6]. Temporal variability of less than one order of magnitude to over three orders of magnitude has been reported for residential buildings [7-9] and less than one order of magnitude to more than two orders of magnitude at commercial and industrial buildings [10-12]. These studies show that temporal variability of indoor air VOCs can often follow a seasonal pattern, with concentrations being highest in the winter months due to the stack effect. Temporal variability can generally be attributed to changes in the building air exchange rate and the soil vapor entry rate, which are, in turn, influenced by environmental and building factors.

Conventional VI assessment and sampling practices are largely based on one model of behavior—one in which the stack effect, driven by indoor/outdoor temperature differentials (e.g., warmer indoor air rising and pulling soil gas), is assumed to control and increase soil gas contaminant concentrations in indoor air [4,5]; thus, the highest concentrations are expected to be observed in “winter”, whether defined based on the calendar or heating equipment use. Conventional VI sampling practices use the same strategy regardless of the climate zone and building type: a limited number of short-duration samples (typically 24 h) collected at dates chosen based on a combination of season (or temperature) and investigator convenience [13].

I&Ts, which include a collection of quantifiable metrics and tools including differential temperature (indoor–outdoor), differential pressure, and indoor air radon concentrations, have been suggested as a potential solution for addressing temporal variability by guiding sampling times and locations as well as making VI pathway assessment and long-term monitoring more informative, efficient, and cost effective [14]. Indicators, such as differential pressure or temperature, help identify an elevated potential for VOC exposures and narrow the assessment period needed to capture reasonable maximum exposure (RME) conditions [14]. Tracers are substances in soil gas (i.e., radon) that migrate from the subsurface to indoor air in a manner similar to that of VOCs of interest, enhance understanding of the conceptual site model, and aid in the identification of conduit pathways. The potential utility of radon as a tracer for VOC vapor intrusion integrating several processes that control attenuation across the building envelope has been widely discussed [15-20]. I&Ts have been demonstrated as low-cost lines of evidence to support future sample collection scheduling and indoor air sampling result interpretation at two residential buildings in Indianapolis, Indiana [8,14-17], and Sun Devil Manor in Layton, Utah [9,14,21-24]. However, both these studies were completed in residential-style buildings in similar temperate climate zones. Another example of a study that applied high temporal resolution measurements of a low-cost air quality indicator to a specific urban area is Lotrecchiano et al. [25].

There have been limited studies of the long-term temporal variability of VI in industrial or commercial buildings. The available studies suggest substantial temporal and spatial variability [10,26,27] and significantly better attenuation factors in large buildings than have been observed in typical residences [28]. Recent work has also highlighted the importance of climate zone and the location of the VOC mass storage relative to the building in controlling the type of temporal variability observed [12,26,29,30]. For example, the temporal variability at commercial buildings in Alaska and New Hampshire has varied from the standard stack effect–driven pattern of higher indoor air VOC concentrations in the winter to higher concentrations being observed in summer, likely due to the source being directly under the slab or in building envelope materials and the climate [26,30]. In the following two limited studies, long-term datasets in multiple heating, ventilation, and air conditioning (HVAC) zones were compared and showed some recognizable effects of heating and ventilation practices on the long-term temporal VOC trends and spatial VOC distributions between zones. The Indianapolis duplex study compared the heated and unheated sides of a residential duplex [21], whereas a study at a Virginia commercial building compared multiple HVAC zones built out as separate walled structures within a warehouse-type space [12]. Neither study identified distinct HVAC patterns associated with indoor air temporal variability on a short repeatable time scale.

Temporal and seasonal variability in indoor air concentrations can also occur due to building ventilation processes that modify building air exchange rates, such as exhaust fans or HVAC systems, as well as wind-driven air leakage [31,32]. For example, exhaust fans force air out of a building, thereby depressurizing and creating a net-negative internal pressure and likely enhancing soil gas entry VI. The stack effect also creates a depressurization in the lower portion of the building and a positive pressurization in the upper portion [32]. However, processes that depressurize the structure and increase the rate of soil gas entry tend also to increase the air exchange rate, so the net effect on indoor air concentration varies with the location of leakage points in the building envelope (walls, roof, slab) and VOC sources [31,33]. Indoor air concentrations can be estimated under various building ventilation conditions by combining three-dimensional building science and VI models [34], but these models are not used in typical VI site assessments.

This paper focuses on the long-term (weeks to seasons) temporal variability in indoor air VOC concentrations at six commercial buildings overlying two adjacent chlorinated solvent contaminated groundwater plumes in Fairbanks, Alaska. The following research questions are explored: (1) How do long-term temporal trends in indoor air VOC concentrations vary across buildings (i.e., what is the influence of building location over different parts of the plume)? (2) What is the influence of HVAC systems and operations on long-term temporal trends in indoor air VOCs? (3) How do indoor air VOCs vary between different HVAC zones within the same building? (4) Are long-term temporal trends in indoor air VOCs predictable in commercial buildings using I&Ts based on current theory (i.e., stack effect vs. soil temperature predominance)?

## 2. Materials and Methods

The research site is located along and in the vicinity of Gaffney Road in Fairbanks, AK. The study site is in a commercial area with a diverse mix of small, medium, and large residential, commercial, and government buildings. The study site is located within two large groundwater chlorinated solvent plumes contaminated with tetrachloroethylene (PCE), trichloroethylene (TCE), and their degradation products cis-1,2-dichloroethene (cDCE), trans-1,2-dichloroethene (tDCE), and vinyl chloride. The two plumes, referred to as the “eastern” and “western” plumes (Figure S1), resulted from historical dry cleaner operations in the area. Each plume is attributed to different former dry cleaner(s). Figure S1 delineates the two plumes (divided by Cushman Street near the source) and shows how they move toward the northwest into neighborhoods with mixed commercial, institutional, and residential buildings.

Since the initial discovery of groundwater contamination by chlorinated VOCs in 1993 [35], several investigations have been performed [36-38]. A summary of the historical groundwater and VI sampling results and conceptual site models (CSMs) is provided in the Supplementary Materials (Table S1, Figure S1). Based on a review of historical studies and CSMs, several test locations identified for this study had been previously tested for VOCs in indoor air or SSSG.

### 2.1. Regional Geology and Climate

Fairbanks is in the interior of Alaska, where the climate is classified as continental sub-Arctic with large temperature variability (i.e., warm summers and very cold winters) and a mean annual temperature just below the freezing point [39]. Absolute maximum daily temperatures up to 36 °C (97 °F) have been recorded in June, and minimum daily temperatures up to −54 °C (−129 °F) have been recorded in January in Fairbanks [40]. Mean annual precipitation for the period from 1916 to 2006 was only 28 cm (11 inches), with most of the precipitation observed in the summer [39]. In winter, monthly average snowfall between 23 and 30.5 cm (9 to 12 inches) is observed between November and March, with maximum daily average snow depths of up to 51 cm (20 inches) in February [41]. During the winter, Fairbanks routinely experiences strong temperature inversions (i.e., temperature increases with elevation) owing to lack of winds, a snow-covered surface, and insufficient sunlight to heat the ground. These are known to be substantial localized variations in weather within the Fairbanks area based on elevation and local topography, sometimes as much as 28 °C (82 °F) during inversions [42]. These inversions can trap pollutants from burning of wood, fuels, and automobiles, including fine particulate matter smaller than 2.5 microns (PM_2.5_), leading to poor air quality [43].

The site is situated on the collective floodplain of the Tanana and Chena Rivers. The surficial geology consists of well-stratified layers of unconsolidated silt, sand, and gravel of the Chena alluvium, which is approximately 91 m (300 ft) thick in river valleys [44]. Test borings conducted within the vadose zone at the site confirmed the presence of alternating layers of sand with varying gravel and silt compositions with the interval near the top of the water table (4.6 m to 5.2 m [15 ft to 17 ft] below ground surface) characterized as poorly graded gravel with sand [36]. Discontinuous permafrost of generally low ice content is characteristic of Chena alluvium sediments, but permafrost in the form of seams and lenses may be found in swale and slough deposits. Where present, permafrost ranges in depth from 0.6 m to 12.2 m (2 ft to 40 ft) below the ground surface [44]. Fairbanks is located in EPA Radon Zone 2 [45], and a 16-square-kilometer hexagon around the site has had historical radon observations between 0 and 34 picocuries per liter (pCi/L) with a mean of 2.57 pCi/L based on 45 observations [46].

The unconfined, alluvial-plain Chena alluvium aquifer can yield significant quantities of water in wells. Recharge to the alluvial-plain aquifer occurs from the Tanana and Chena Rivers, with a relatively small amount resulting from infiltration of precipitation. Groundwater levels in the alluvial-plain aquifer respond relatively quickly to increases in the stages of the Tanana and Chena Rivers. Wells completed in the alluvial-plain aquifer within half a mile of either river show the greatest elevation increases because of increased river flow [47]. Data gathered during previous groundwater assessments at Gaffney Road East and Gaffney Road West indicate that groundwater in the unconfined alluvial-plain aquifer is shallow at about 6.1 m (20 ft) below ground surface, with flow generally toward the northwest [36].

### 2.2. Building and HVAC Characteristics

Six commercial building structures ranging from 149 to 6500 m^2^ (1600 and 70,000 ft^2^) were selected for this study (Table 1). These structures include four buildings over the eastern plume and two buildings over the western plume. Building features and characteristics were recorded during site visits and walk-through inspections on survey forms similar to those recommended in the EPA VI Guidance [48]. The survey forms detail or confirm the layout, construction (e.g., slab-on-grade, crawl spaces), potential VOC sources (e.g., cleaning products, VOC sinks such as carpets, furniture, and draperies), and operating processes (type of heating or cooling system, etc.) of the units that may influence contaminant entry. Primary building characteristics are given in Table 1. Significant changes in the routine position or settings of interior and exterior doors, windows, and HVAC systems were documented on data collection forms and summarized in an event log.

Larger commercial structures can have multiple HVAC and sampling zones (offices, factory floors, etc.). HVAC zones (thermal zones) are spaces controlled with one thermostat typically served by a single air handling unit (Table 1). Sample zones are enclosed locations in a building where at least one indoor air sample has been collected. Ideally, a sample zone would have limited air mixing with air from other sample zones. The HVAC or sample zones of primary interest to this study are those nearest the points of entry of soil gas into the building. Therefore, this study focused primarily on the basement (if present) and first floor, which were expected to be at least two separate zones. Additional zones within the studied buildings were identified through a visual and interview-based survey process.

Building characteristics of each study building are individually described in the Supplementary Materials. It is important to note that during the study period, the building was operated as realistically as possible, with the operations controlled by the regular business or residential occupants.

### 2.3. Weather

Meteorological measurements at the study site were made using an on-site weather station at the residential-style office and supplemented with data from the closest National Weather Service facility located at the Fairbanks International Airport (PAFA), located approximately 9.7 km (6 mi) west/southwest from the site. The on-site weather station used for this study was the RainWise MK-III system (RainWise, Boothwyn, PA, USA), which records ambient temperature (T), relative humidity (RH), dewpoint T, wind speed and direction, barometric pressure, and rainfall. The on-site weather station experienced frequent outages because it shared the computer network resources of the residential-style office; therefore, results from this system were intercompared with the professional airport weather station and generally showed good correlation.

### 2.4. Sampling and Analysis

#### Indoor and outdoor air VOC samples

Indoor and outdoor air were monitored using diffusive sorbent samplers (Radiello^®^, RAD145, Sigma-Aldrich, Inc., St. Louis, MO, USA). Samples were normally collected over a 1-week period, although durations as short as 6 h were seen in intensive sampling events. Analysis was performed following EPA Compendium Method TO-17 [48] using gas chromatography/mass spectrometry (GC/MS) for PCE, TCE, cDCE, and tDCE. An initial calibration was performed, and continuing calibration verification (CCV) samples were run at the beginning and end of each analytical batch. Corrective action (typically preparing a new CCV standard or performing a new initial calibration) was taken as soon as possible when the target compound recovery was outside of the project QC criteria of 70–130% or the internal standard recovery was outside of 60–140%. The indoor and outdoor air, subslab vapor, and soil vapor monitoring locations in each building are summarized in Table S1.

#### Indoor and outdoor air VOCs—continuous monitoring by gas chromatograph/ electron capture detector (GC/ECD).

Continuous VOC data were collected at the church and residential-style office using two separate GC/ECD instruments. Analysis was performed following EPA Compendium Method TO-14A [49]. An initial calibration was performed, and continuing calibration verification samples were run one to two times per day. Corrective action (typically preparing a new verification standard or performing a new initial calibration) was taken as soon as possible when drift beyond 70–130% recovery was noted. A GC-ECD control chart is provided in the Supplementary Materials (Figure S4a-d). The GC sample introduction systems were configured with two separate multiport valves on each instrument. Indoor and outdoor air samples were collected through the primary valve (A) and subslab vapor and soil vapor through the secondary valve (B) to limit cross-contamination potential for each matrix. Teflon tubing was run from the various sampling locations to the GC/ECD instruments. Vacuum pumps pulled samples through the tubing and into the instruments’ sample loops before analysis.

The PCE concentration of samples is the focus of this paper as the majority of results for TCE, cDCE, and tDCE were at or near the reporting limit. The GC/ECD instruments achieved a reporting limit of 0.1 part per billion by volume (ppbv), or 0.7 micrograms per cubic meter (μg/m^3^) for PCE. In the case of indoor air PCE, estimating the instrument’s method detection limit based on a 3:1 signal-to-noise ratio yielded a value of about 0.02 ppbv, which is equivalent to about 0.14 μg/m^3^. Indoor, outdoor, and floater samples were analyzed every 2 to 3 h. Subslab vapor and soil gas samples were analyzed every 6 to 13 h, producing two to three samples per day.

#### Indoor air radon.

Radon was monitored using a consumer-grade radon detector: RadonEye^®^ RD200 (Ecosense Inc., San Jose, CA, USA). This unit has good sensitivity, agreement with certified devices, and hourly readability [50,51].

#### External soil gas and subslab probe construction.

Four external soil gas probes were installed outside the Church on the north, south, east, and west sides of the building (Figure S3a,b). It is important to note that the depth of the church basement is approximately 2.4 m (8 ft) below ground surface.

Each location contained nested probes at four depths below ground surface (bgs): 0.6 m (2 feet [ft]), 1.4 m (4.5 ft), 2.1 m (7 ft), and 2.9 m (9.5 ft), providing information on the soil gas available to infiltrate through the basement floor and walls. Soil gas probes were constructed in accordance with Final Quality Assurance Project Plan (QAPP) [52-54] and field screened using a photo ionization detector. All four nested probe locations were completed with concrete or cold patch asphalt and a flush mount well cap and their locations were surveyed. Approximately 10 days following installation, leak checks were performed at all four probe locations using the tracer gas/shroud method [55]. Soil gas probes at all depths and all locations passed the leak checks and were clear of obstructions prior to sample collection.

#### Exterior air and SSSG radon.

The DURRIDGE RAD7 (Durridge Company, Inc., Billerica, MA, USA) portable instrument was used for on-site radon analysis of outdoor air and subslab grab samples. In addition to the radon concentration in air, the RAD7 measures and records ambient temperature and relative humidity, with the relative humidity reading used to ensure accuracy of the measurement. Outdoor air samples were collected through a heated sample line. Instrument setup and operation were performed in accordance with the manufacturer ’s instructions and with EPA guidelines for using continuous radon monitors [5].

#### Subslab and exterior soil gas VOCs.

VOCs in soil gas were determined by modified Method TO-17 [48] using thermal desorption. A measured volume (50 mL to 100 mL) of soil vapor was pulled through a sorbent tube using a calibrated gas-tight syringe. Stainless steel thermal desorption tubes with dimensions of 8.9 cm (3.5 in) in length by 0.63 cm (0.25 in) in outside diameter, packed with approximately 0.2 g of Tenax_™_ TA (Markes International, Inc., Sacramento, CA, USA), were used for sample collection.

## 3. Results and Discussion

In this article, we compare the long-term PCE and radon trends in a series of independent buildings and HVAC zones in those buildings experiencing the same weather conditions. The buildings, however, differ in their location, with respect to the PCE source (environmental release); style of construction; current occupancy; and heating and cooling systems. In this paper, we use a visual pattern recognition/descriptive approach to analyze the time series data. A more formal time series analysis of multiple VI datasets will be presented in a companion paper.

**Figure 1.**
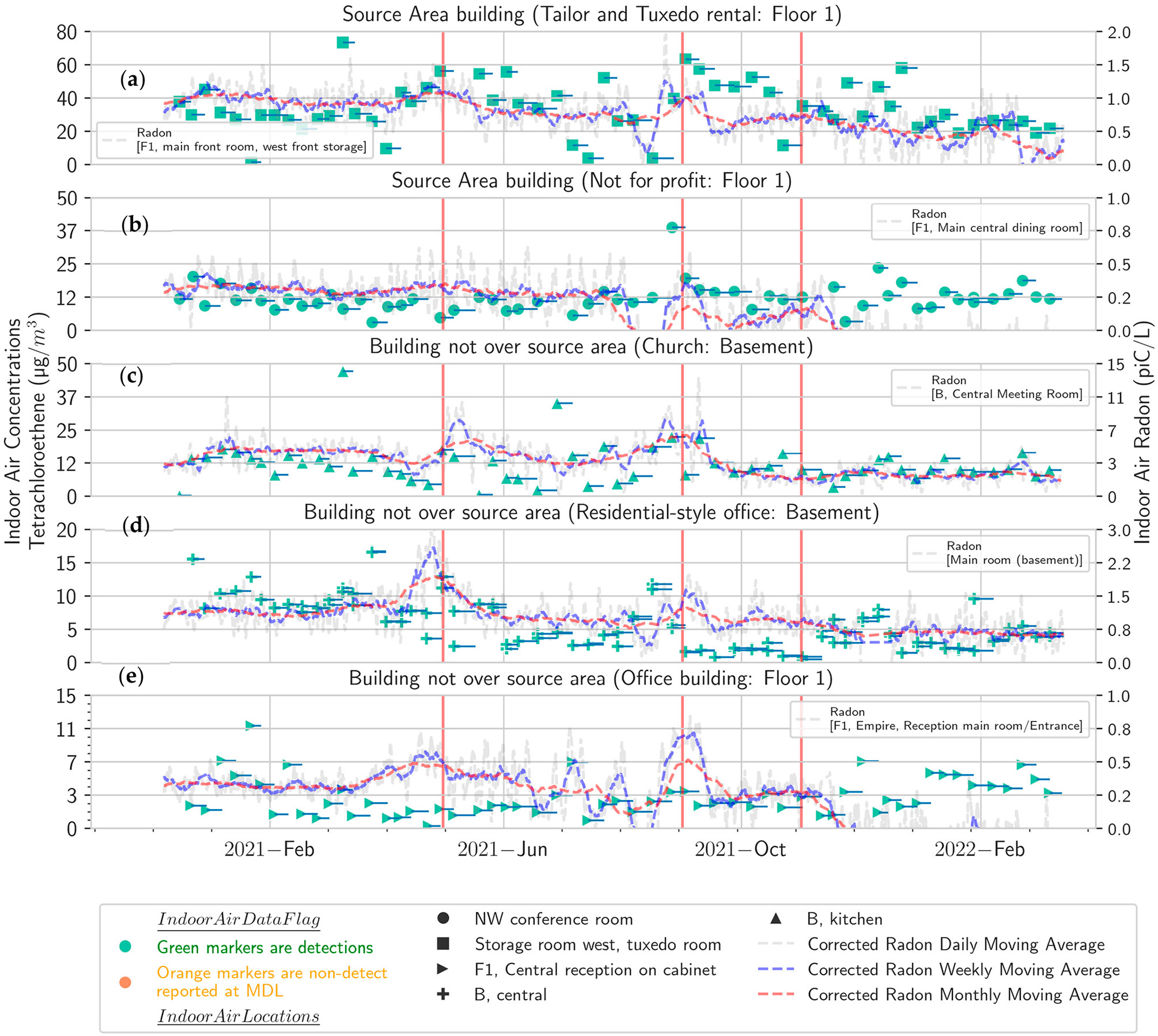
Measured PCE and radon times series plots stacked by building and floor (**a**) tailor and tuxedo rental shop, (**b**) not-for-profit, (**c**) church, (**d**) residential-style office, (**e**) office. Sample durations for each VOC sample (green markers) are shown as blue lines. Red vertical lines show common features in the radon plots across the buildings.

There are some common features in the radon plots across many of the buildings, such as peaks in May, September, or November 2021 (red vertical lines in Figure 1). Other common features are seen in many of the PCE plots as well from buildings away from the source area (church, residential-style office, and office building), such as lower concentrations in summer 2021 (June, July, early August). The summer low on the upper floor of the church is particularly pronounced because a window was kept open during that season (Figure S6b). Those buildings away from the source area tended to exhibit peaks in late spring and early fall. The buildings overlying the source area (a not-for-profit and a tailor and tuxedo shop) showed a more uniform seasonal PCE distribution with some indication of a summer high. Thus, the general pattern of spring and fall peaks appears to be common at this site for PCE and radon.

The observation that the buildings over the source area showed a more uniform seasonal distribution than the buildings at a further distance from the source mass could be attributable to two mechanisms. First, in buildings over the soil source area, the transport pathway for advective VOC entry is short, and thus, vapor entry can occur during brief periods of building depressurization. In contrast, further downgradient, the migration pathway from groundwater is longer, and thus, a more sustained period of advective flow may be needed to overcome the sorptive capacity of shallow soils. A second mechanism is that close to the building, a diffusive mechanism through the foundation or out of the concrete slab itself may be important in some structures over high concentration sources [26,56,57]. The diffusive mechanism could be less seasonally controlled than the advective mechanism.

**Figure 2.**
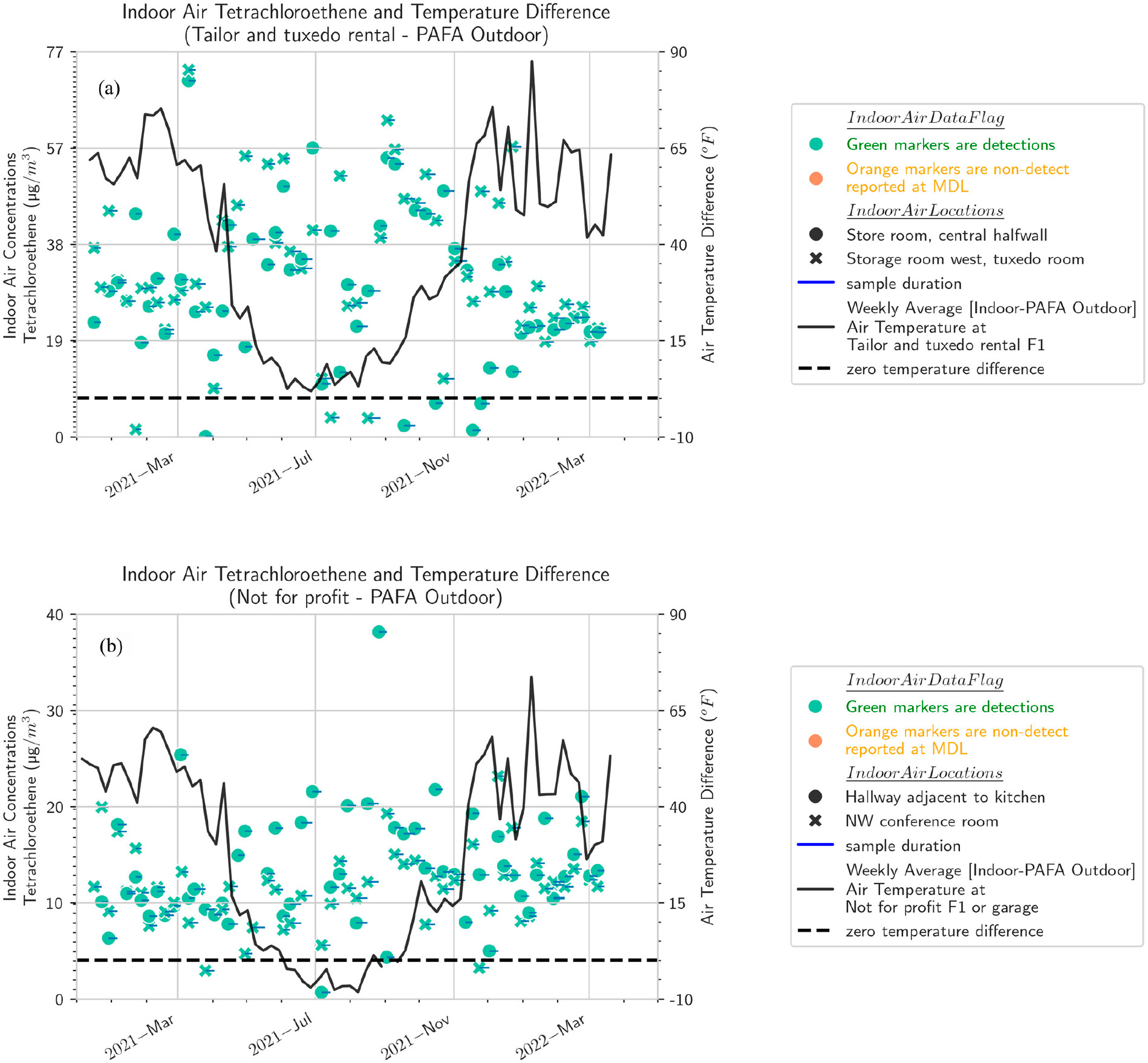

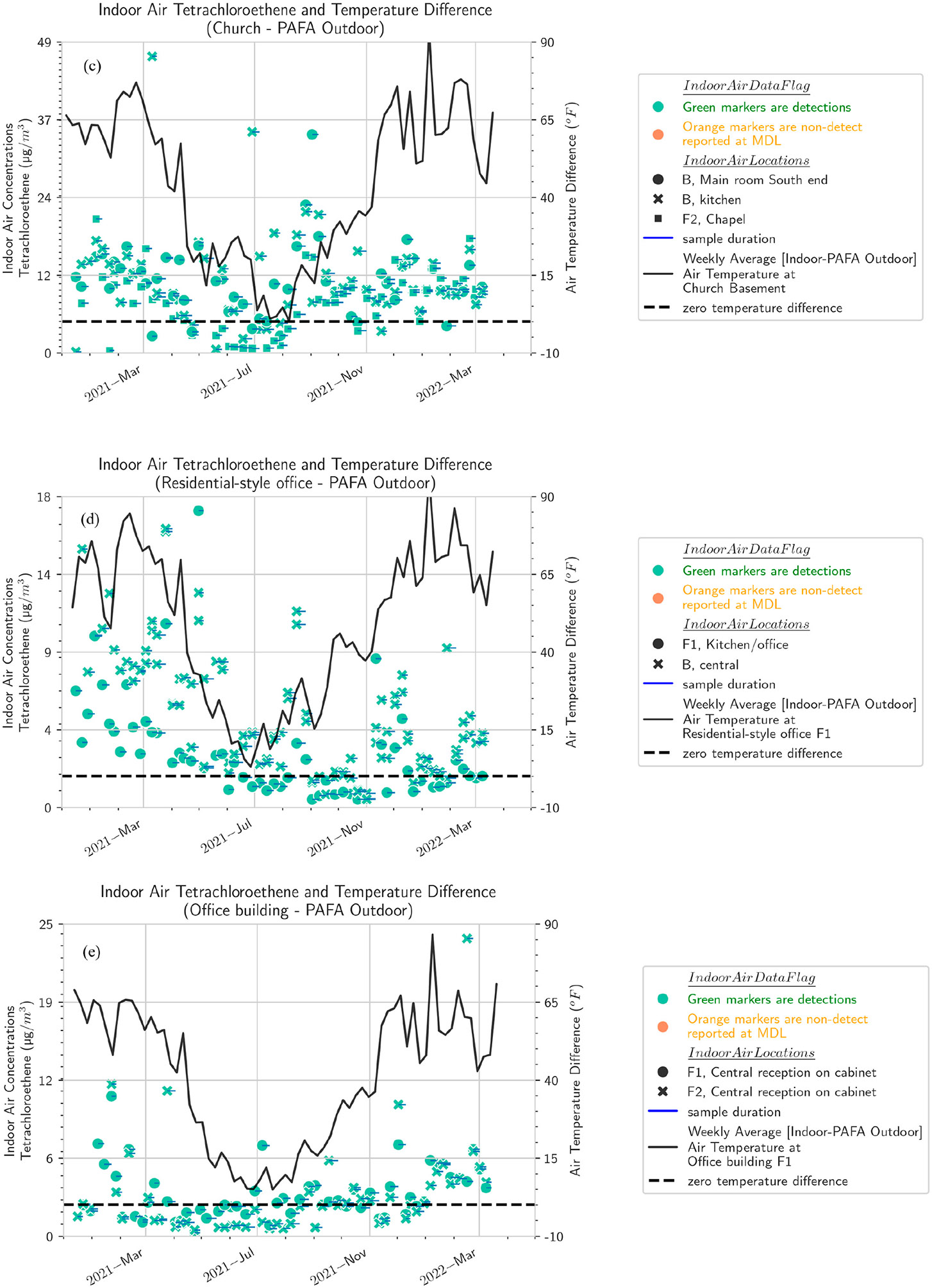
Weekly averaged temperature differential between indoor air and outdoor air (measured at PAFA) along with indoor air PCE concentrations by building [(**a**) tailor and tuxedo rental shop, (**b**) not-for-profit, (**c**) church, (**d**) residential-style office, (**e**) office]. Sample durations for each VOC sample (green markers) are shown as blue lines.

The indoor/outdoor temperature differential is a measure of the driving force for soil gas entry and air exchange provided by the stack effect. The temperature differentials in Fairbanks are, as expected, much larger than those at the other sites that have been intensively studied in the vapor intrusion literature in Indiana, Utah, and Virginia. Temperature differentials are calculated from indoor temperatures measured in each individual building but are broadly similar across buildings because the outdoor temperatures are common, and most buildings have similar thermostatically controlled indoor temperatures. Figure 2a-e show that across the five buildings, there is substantial visual correlation between the seasonal trend in differential temperature and PCE concentrations. This result suggests that the stack effect mechanism is influential in these buildings. This result also shows that the increase in the soil gas entry rate driven by the stack effect outweighs the influence of the stack effect in increasing building air exchange. Differential temperatures across all buildings are plotted in Figure S6, with time intervals ranging from 6 h to a weeklong average. There is a long period from about November 2021 to April 2022 in which the seasonal component of the differential temperature is essentially steady, but there is as much as 15 to 27 °C (60 to 80 °F) of diurnal or short-term variation in the differential temperature. This pattern is consistent across buildings.

In this climate, it is only in basements that the differential temperature becomes negative during the summer; thus, a reverse stack effect would not be a major factor in VI temporal variability at this site. Essentially, there is little need for mechanical cooling in this climate, and natural ventilation is adequate in the summer.

This dataset also allows us to assess whether the conventional “winter worst” stack effect is the primary control on vapor intrusion in these buildings or if soil temperature is the primary control on VI [58] with some stack effect contribution, as previously hypothesized by Barnes and McRae [26] in Fairbanks, Alaska. This current study includes buildings near the source of the Gaffney East plume, as well as buildings connected to the Gaffney source areas (near the point of original release) through vadose zone, groundwater, or sewer transport.

**Figure 3.**
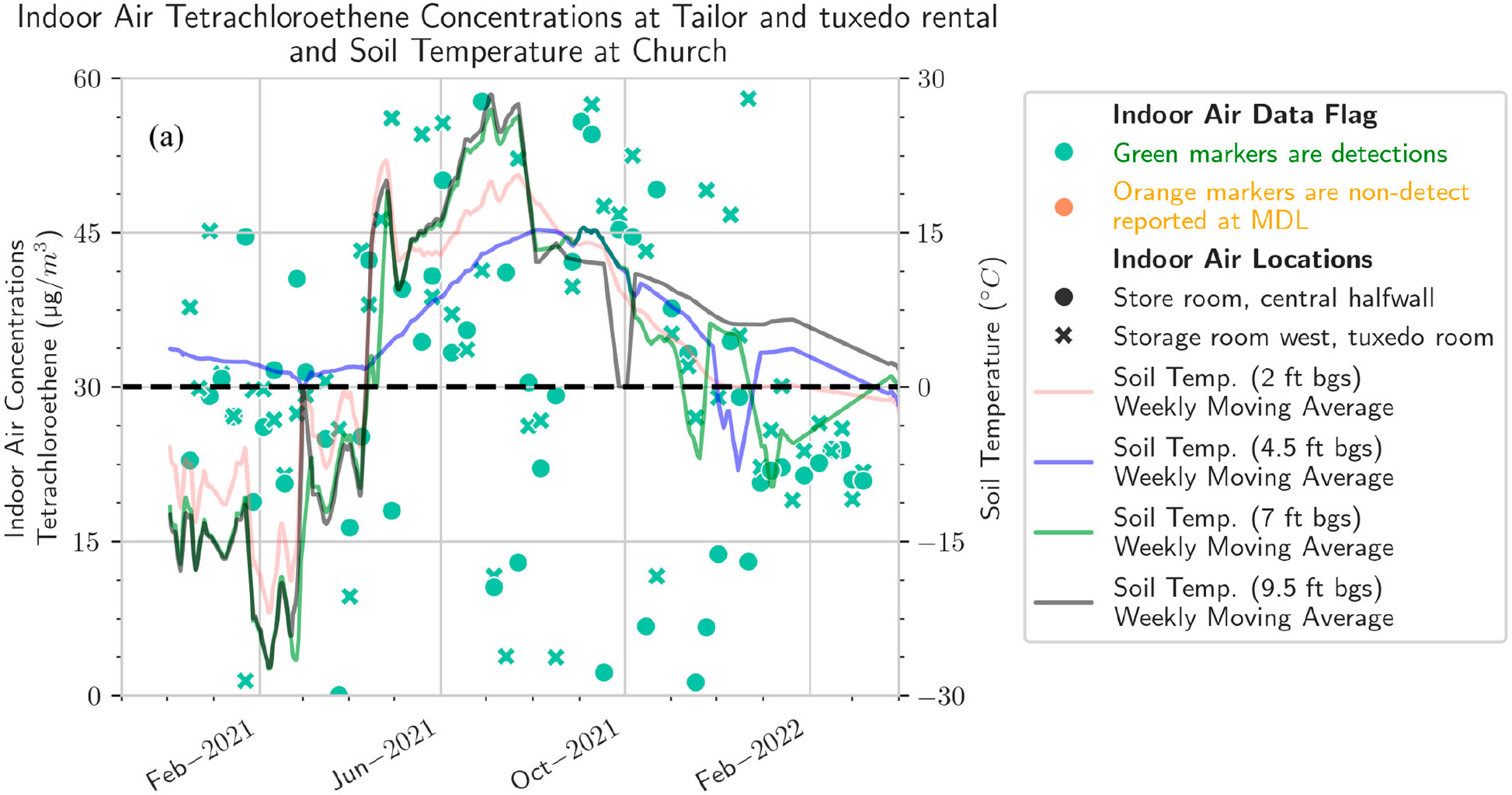

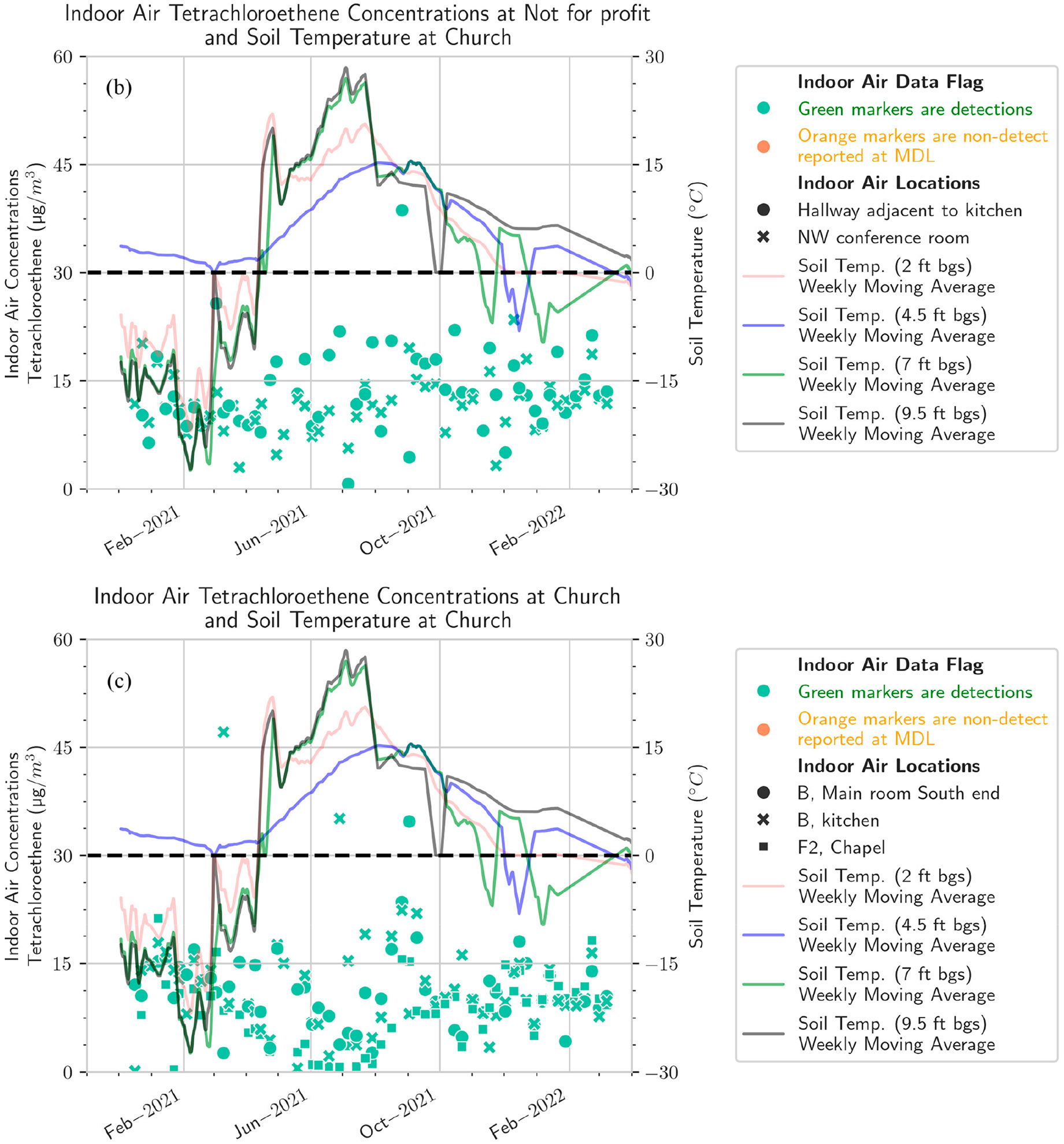

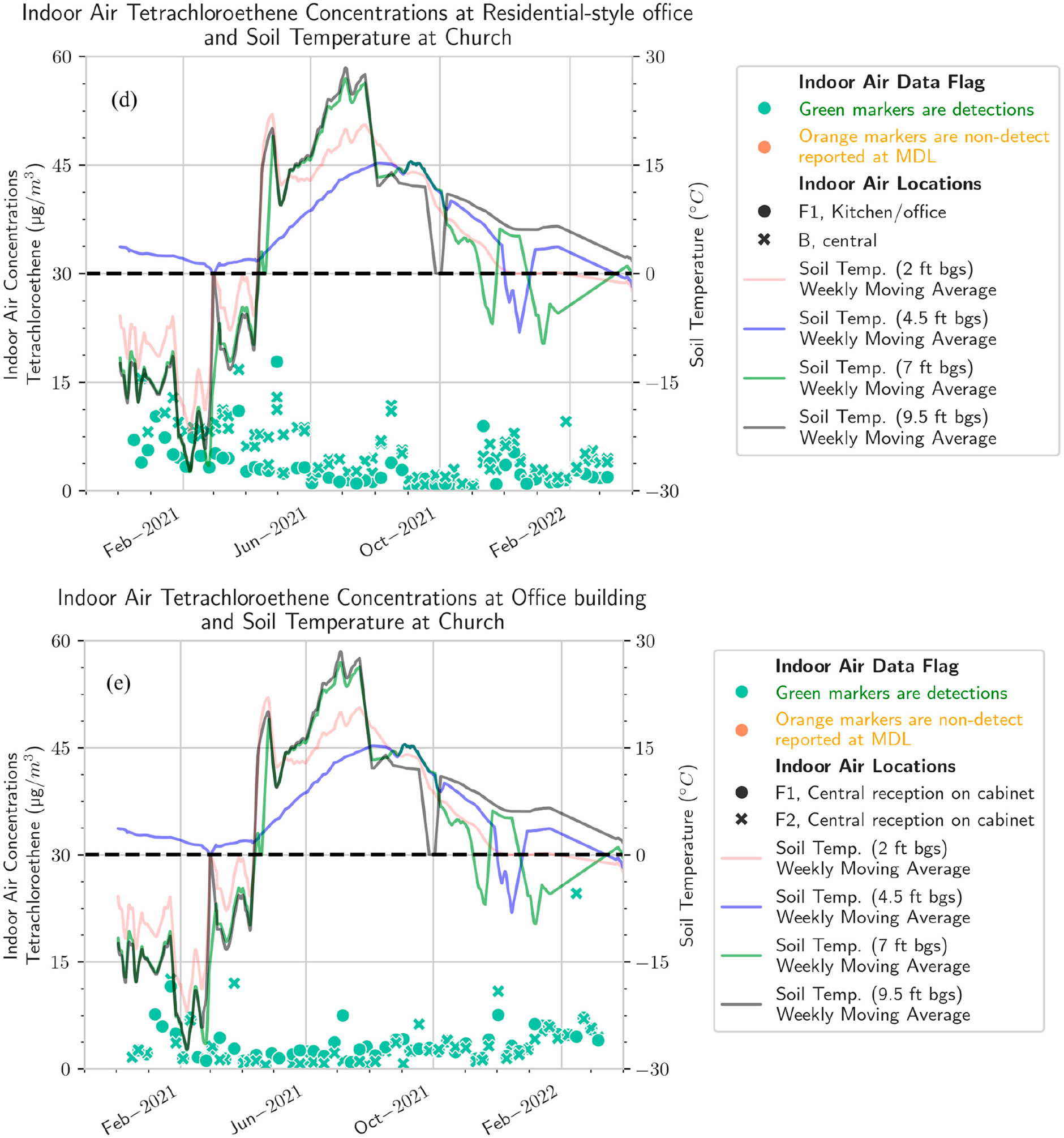
Indoor air concentrations of PCE, by building [(**a**) tailor and tuxedo rental shop, (**b**) not-for-profit, (**c**) church, (**d**) residential-style office, (**e**) office)] compared to soil temperature at church.

The results show that soil temperature is not the dominant factor in indoor concentrations in buildings that are not over the source area (Figure 3). However, the VOC data pattern for the source area buildings (not-for-profit and tailor and tuxedo shop) shows a summer high pattern that is more consistent with a soil temperature–dominated behavior.

**Figure 4.**
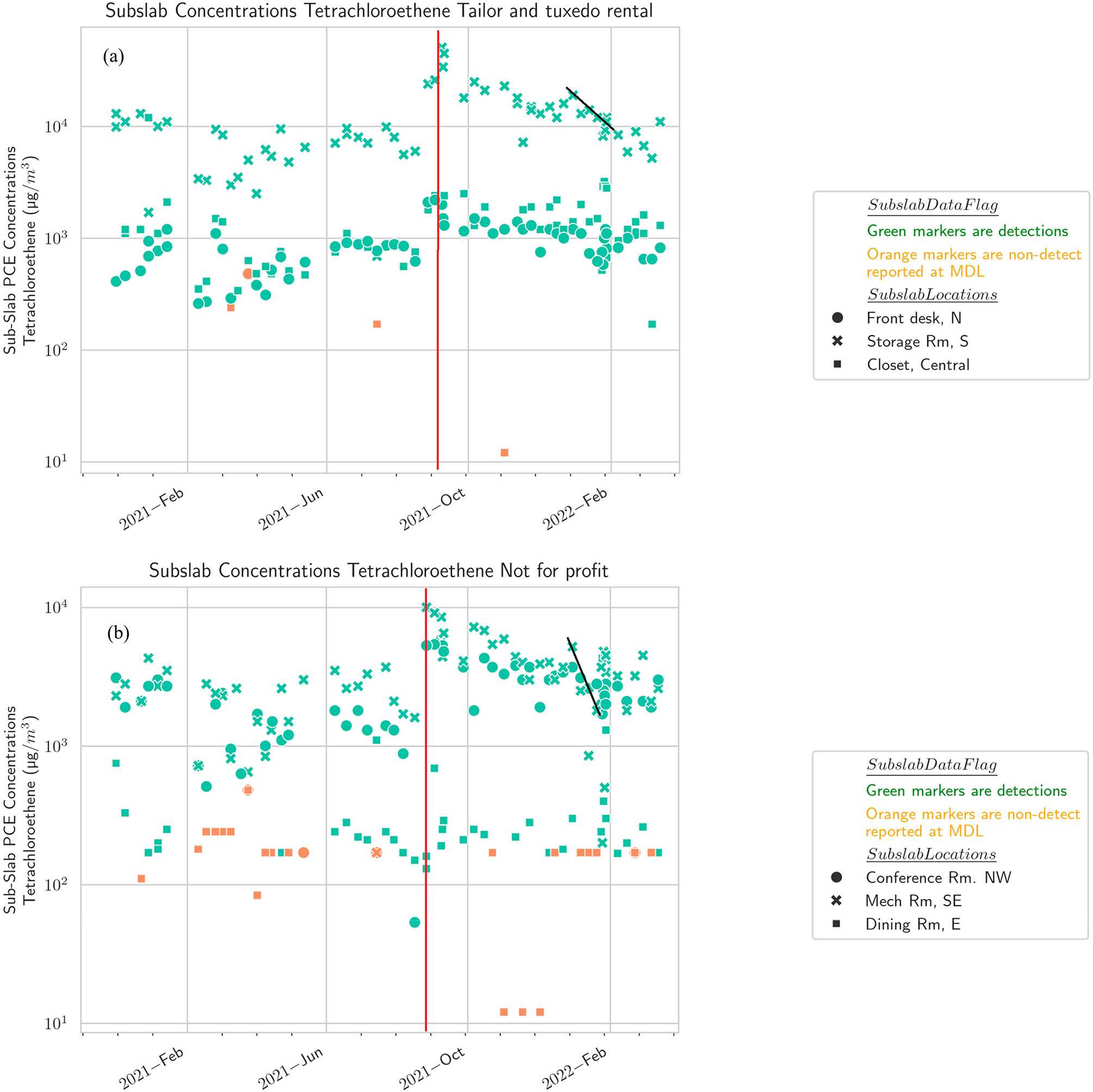

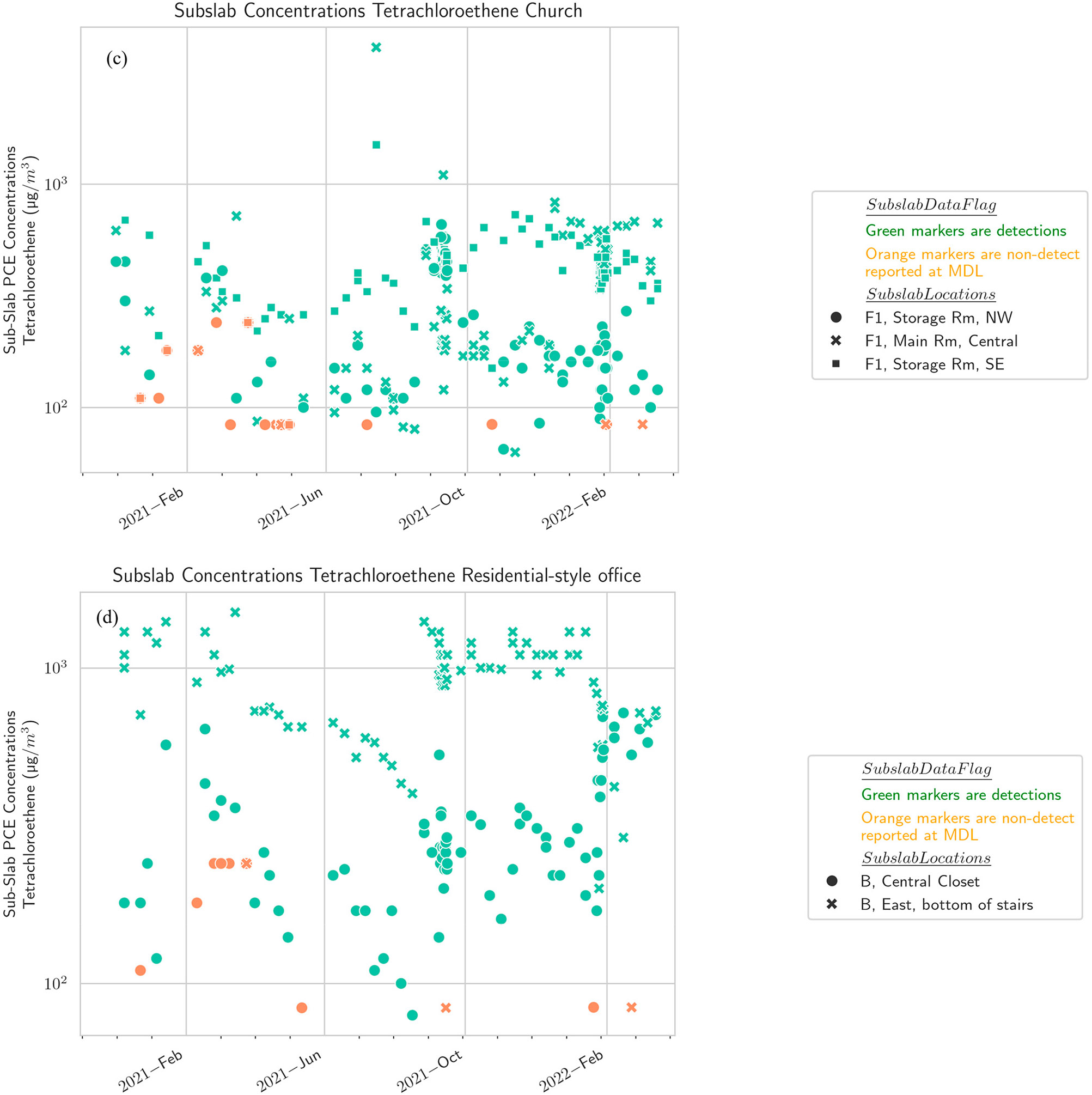

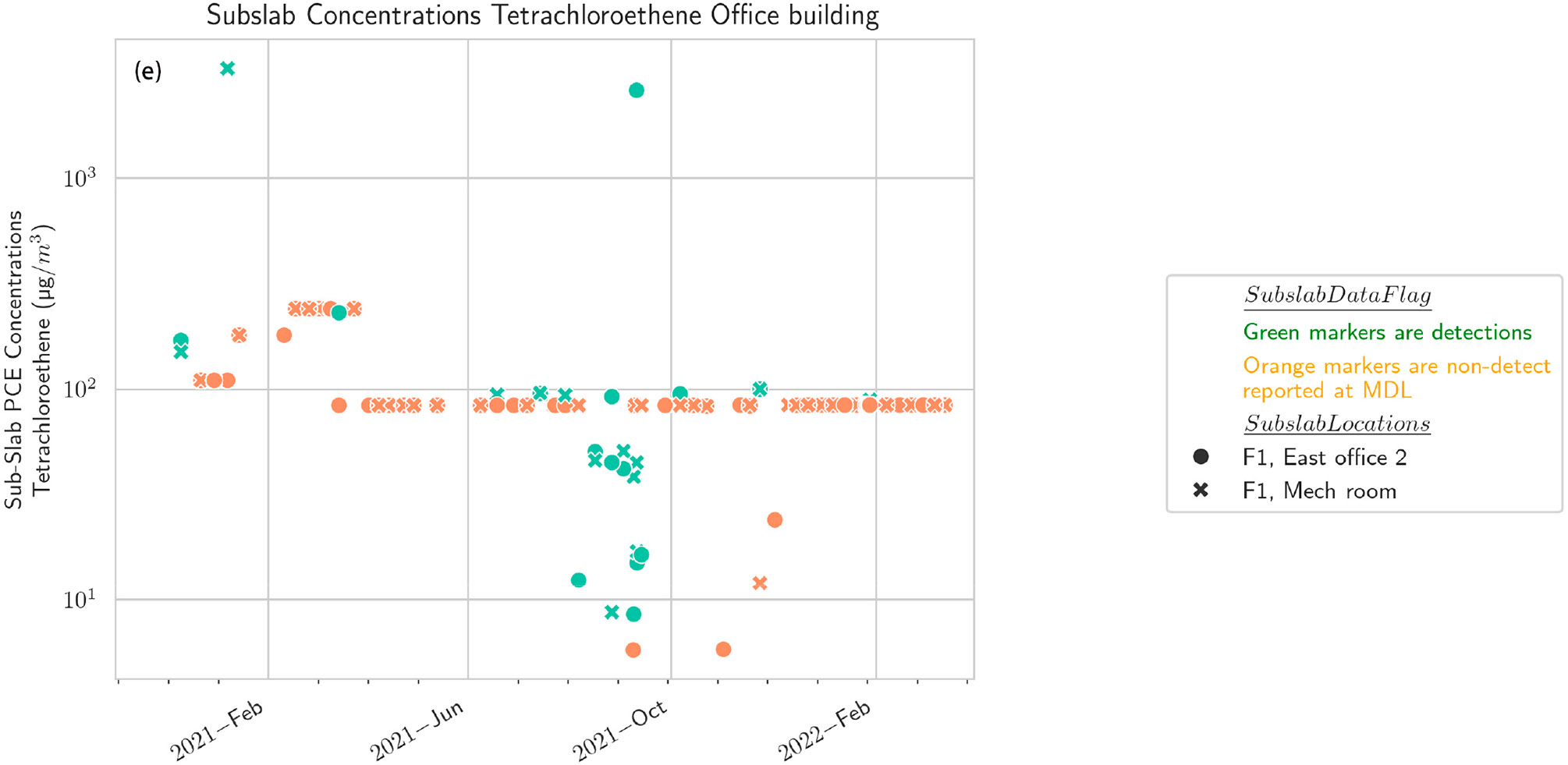
Subslab concentration versus time, by building [(**a**) tailor and tuxedo rental shop, (**b**) not-for-profit, (**c**) church, (**d**) residential-style office, (**e**) office)]. A strong peak in subslab PCE in early September at the not-for-profit and tailor and tuxedo rental buildings is shown as a red line and a less pronounced dip in January is shown as a black line.

**Figure 5.**
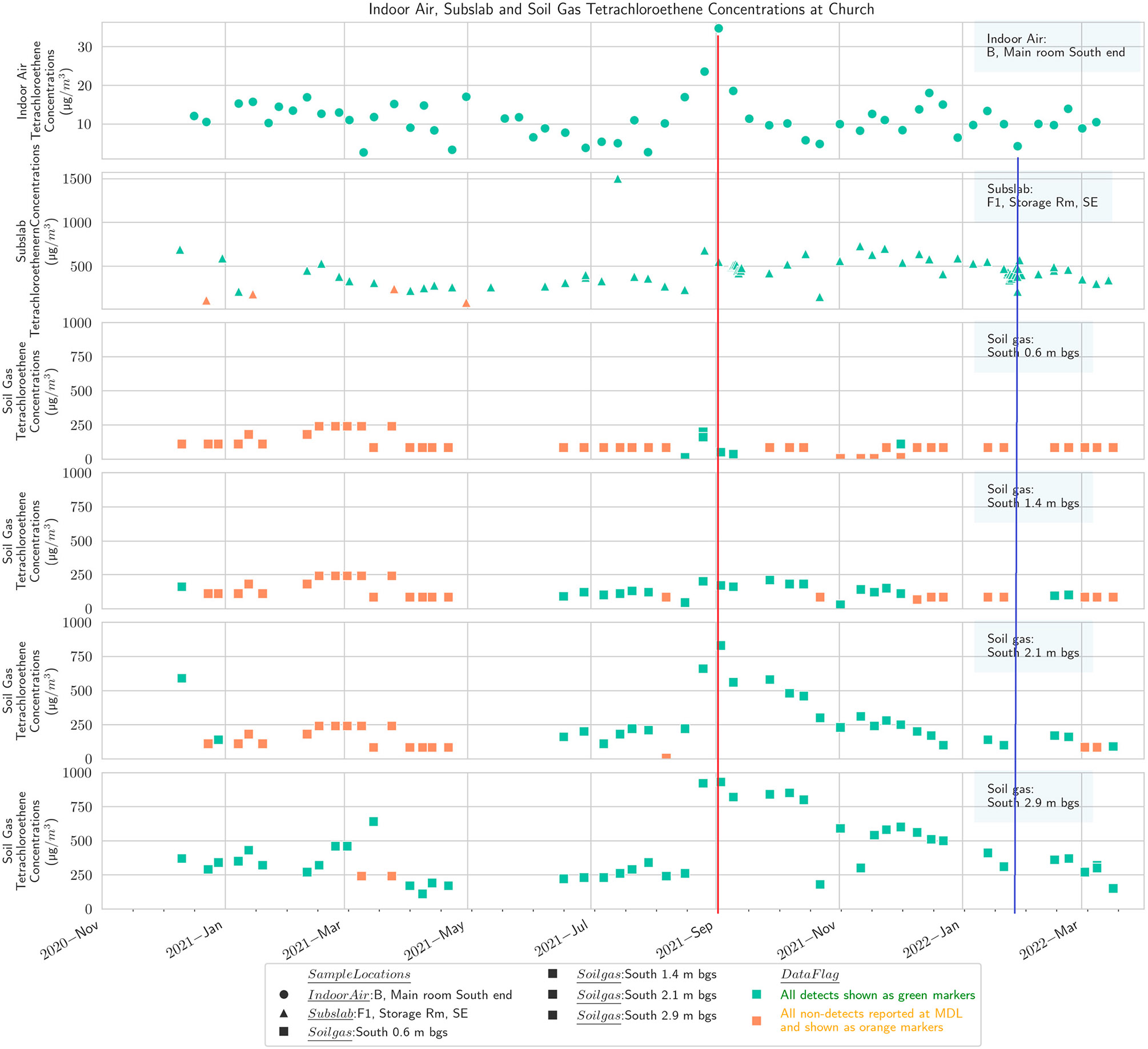
Multidepth soil gas PCE (south), subslab, and basement indoor air at the church. Red line shows maximum in September, and blue line shows minimum in winter.

For the two adjacent buildings over the source area (not-for-profit: 1995 μg/m^3^ average subslab PCE; tuxedo and tailor shop: μg/m^3^ 4711 average subslab PCE), the average PCE and radon concentrations in SSSG between the buildings are within two to three times of each other. The seasonal pattern of subslab PCE exhibits a strong peak in early September (Figure 4, red line) and a less pronounced dip in January (Figure 4, black line). The estimated yearlong radon attenuation factor is 0.0012 for the not-for-profit and 0.0040 for the tuxedo and tailor shop. The PCE attenuation factor from subslab to indoor air was calculated on a month-by-month basis and ranged from 0.003 to 0.147 for the not-for-profit and 0.007–0.081 for the tuxedo and tailor shop (Table 2, Figures S8-S11). The not-for-profit building has a modern hot air circulation heating system, whereas the tuxedo and tailor shop has baseboard heating without hot air circulation. The seasonal pattern of indoor PCE, however, is similar between the two buildings (Figure 5), suggesting that the presence or absence of forced air circulation is not the primary control on the seasonal pattern.

To understand this seasonal indoor air behavior in more depth, we examined the spatial and then temporal patterns of the soil gas results in two buildings more distant from the source, which had, on average, lower subslab PCE concentrations (church: 339 μg/m^3^; residential-style office: 587 μg/m^3^).

### 3.1. Church—PCE

**Figure 6.**
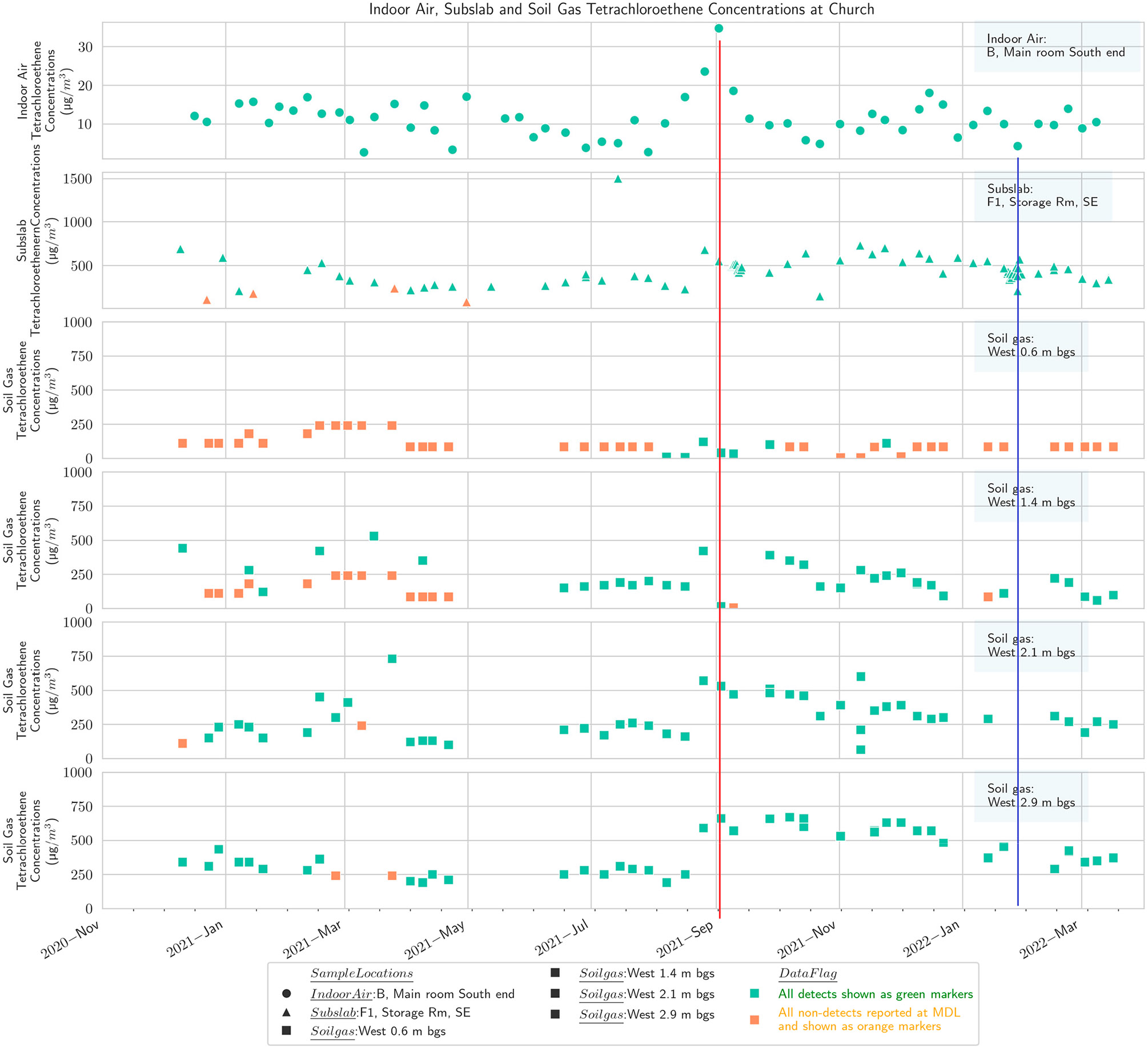
Multidepth soil gas PCE (west), subslab, and basement indoor air at the church. Red line shows maximum in September, and blue line shows minimum in winter

**Figure 7.**
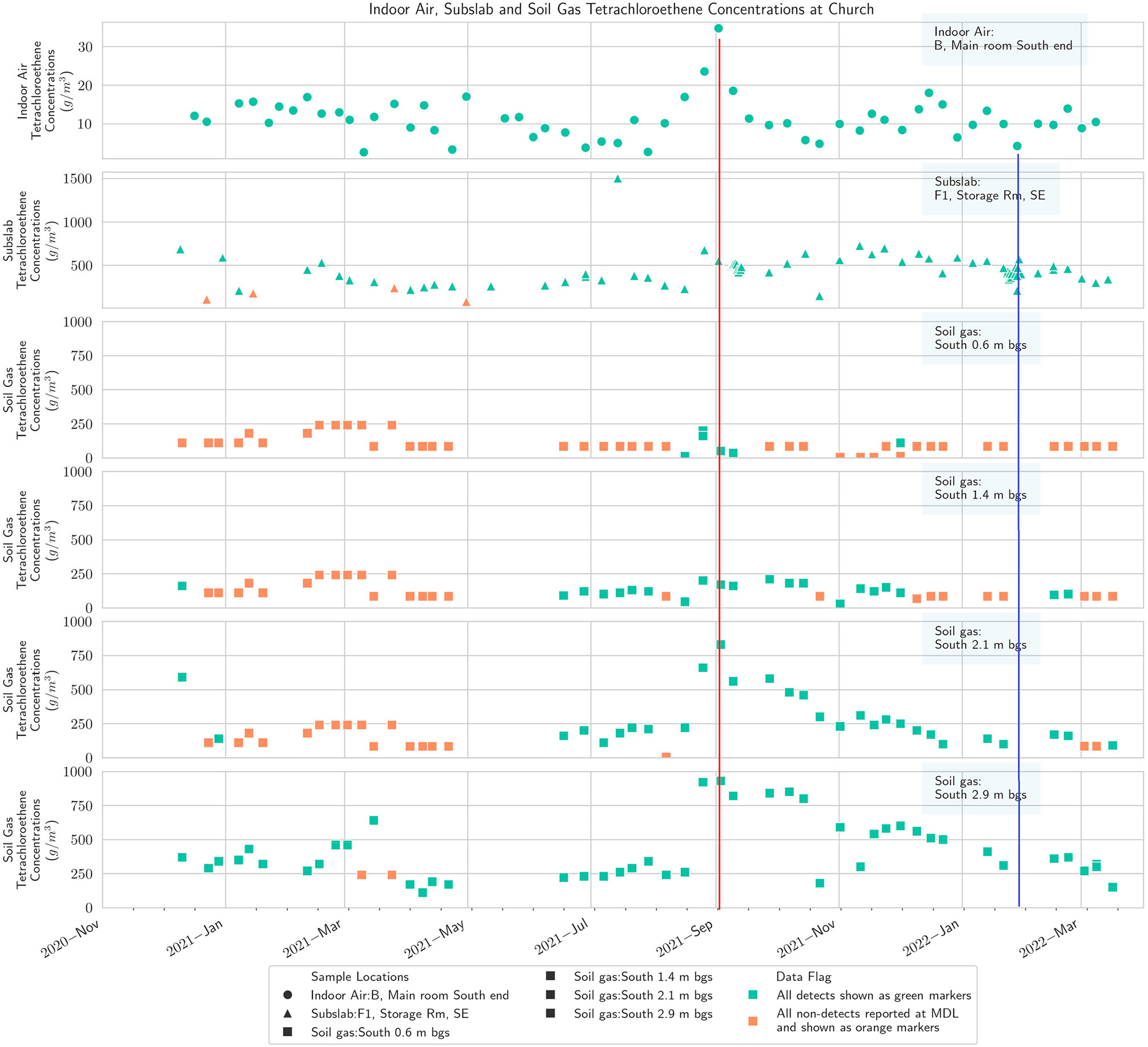
Multidepth soil gas PCE (east), subslab, and basement indoor air at the church. Red line shows maximum in September, and blue line shows minimum in winter.

**Figure 8.**
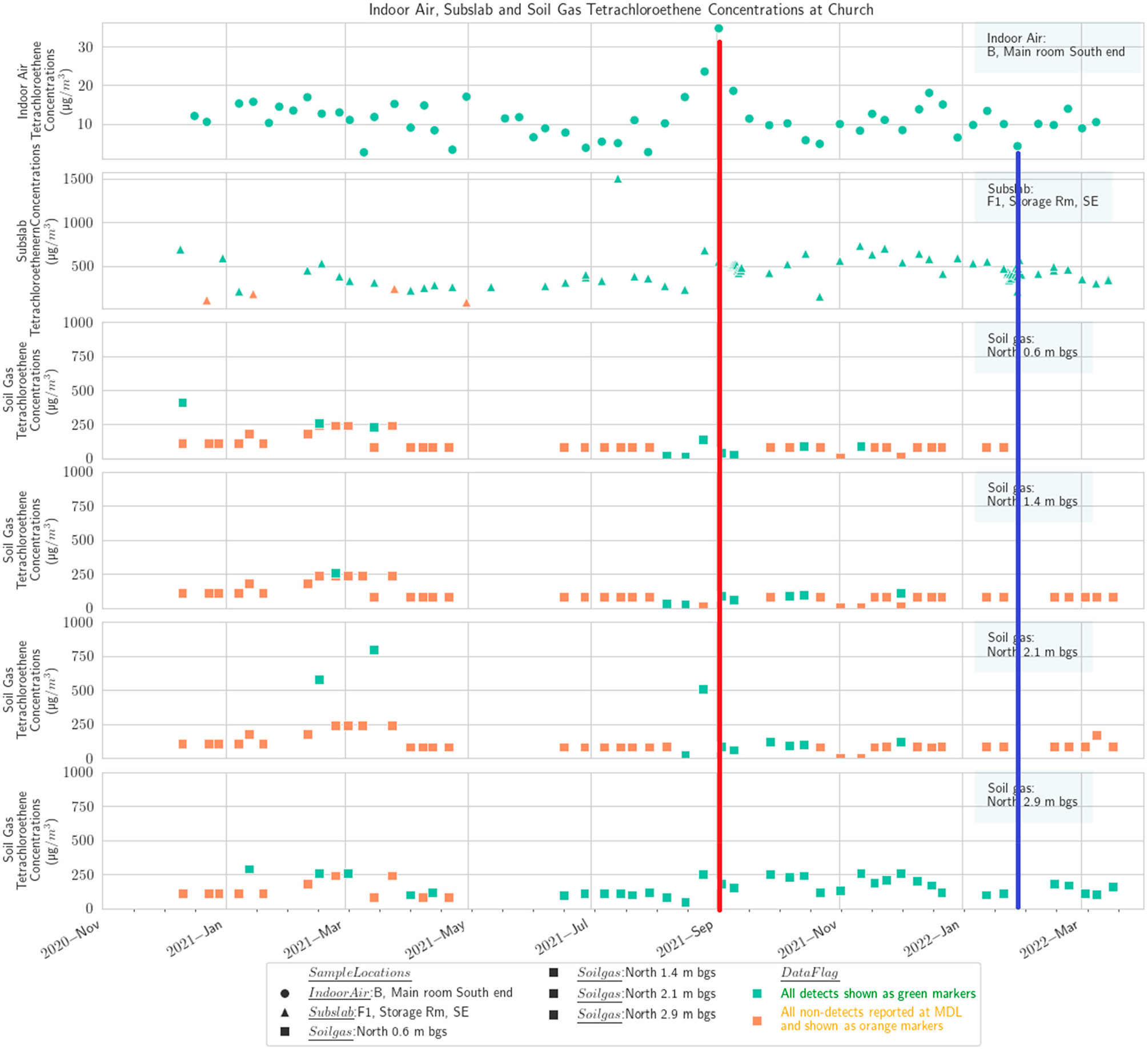
Multidepth soil gas PCE (north), subslab, and basement indoor air at the church. Red line shows maximum in September, and blue line shows minimum in winter.

Results from the multidepth external soil gas clusters on four sides of the church building at four depths, 0.6 m, 1.4 m, 2.1 m, and 2.9 m (2 ft, 4.5 ft, 7 ft, and 9.5 ft), are presented in Figures 5-8, along with concentrations from subslab ports and the indoor air in the church basement. To the extent feasible, the indoor air sampling locations and subslab locations in the church portrayed on these figures are selected to spatially align with the location of the external soil gas clusters.

The results show that PCE concentrations in soil gas generally decrease at shallower depths, as would be expected from a standard groundwater source based VI CSM (Figure S2) and prior experience at other sites [59,60]. There is substantial spatial variability, with the concentrations on the north side of the building being lower, which would not necessarily have been assumed based on the generalized plume shape and building position (Figure S3a,b). The previously available delineation information would not have been fine enough resolution to suggest one side of the church as having a markedly higher concentration than the other.

The PCE temporal pattern in soil gas at the church broadly matches the IA temporal pattern discussed earlier with a September maximum [59]. The conjunction of highest annual soil temperature in late summer with the initiation of heating system operation in September was hypothesized by Barnes and McRae [26] to explain peak indoor concentrations in September. September also corresponds to a maximum high point in groundwater levels in our study (Figure S9), which could displace soil gas upward and shorten the distance between VOC source and building entry points. The data from the west and north soil gas clusters outside the church also show a peak in March, corresponding to indoor air observations in other buildings at the site during this study [59]. However, a prominent peak in the church indoor air was generally not observed except in one of the three indoor locations routinely sampled, the kitchen on the north end of the building (Figure 8).

### 3.2. Residential-Style Office Building—PCE

**Figure 9.**
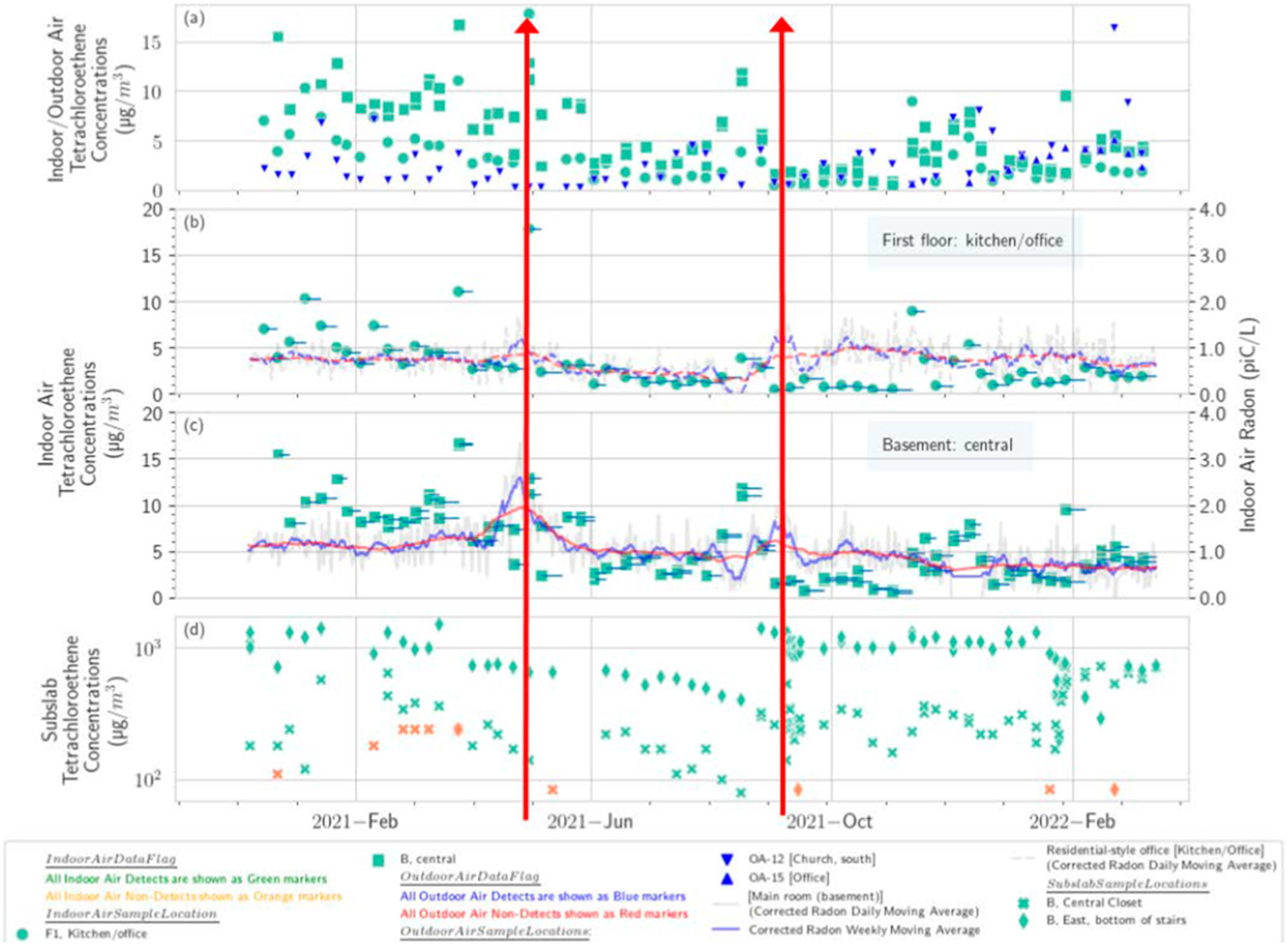
Residential-style office: PCE in indoor air (**a**); PCE and radon in basement (**b**); PCE and radon on first floor (**c**); and subslab soil gas (**d**). Red lines show peaks in concentration across the building.

**Figure 10.**
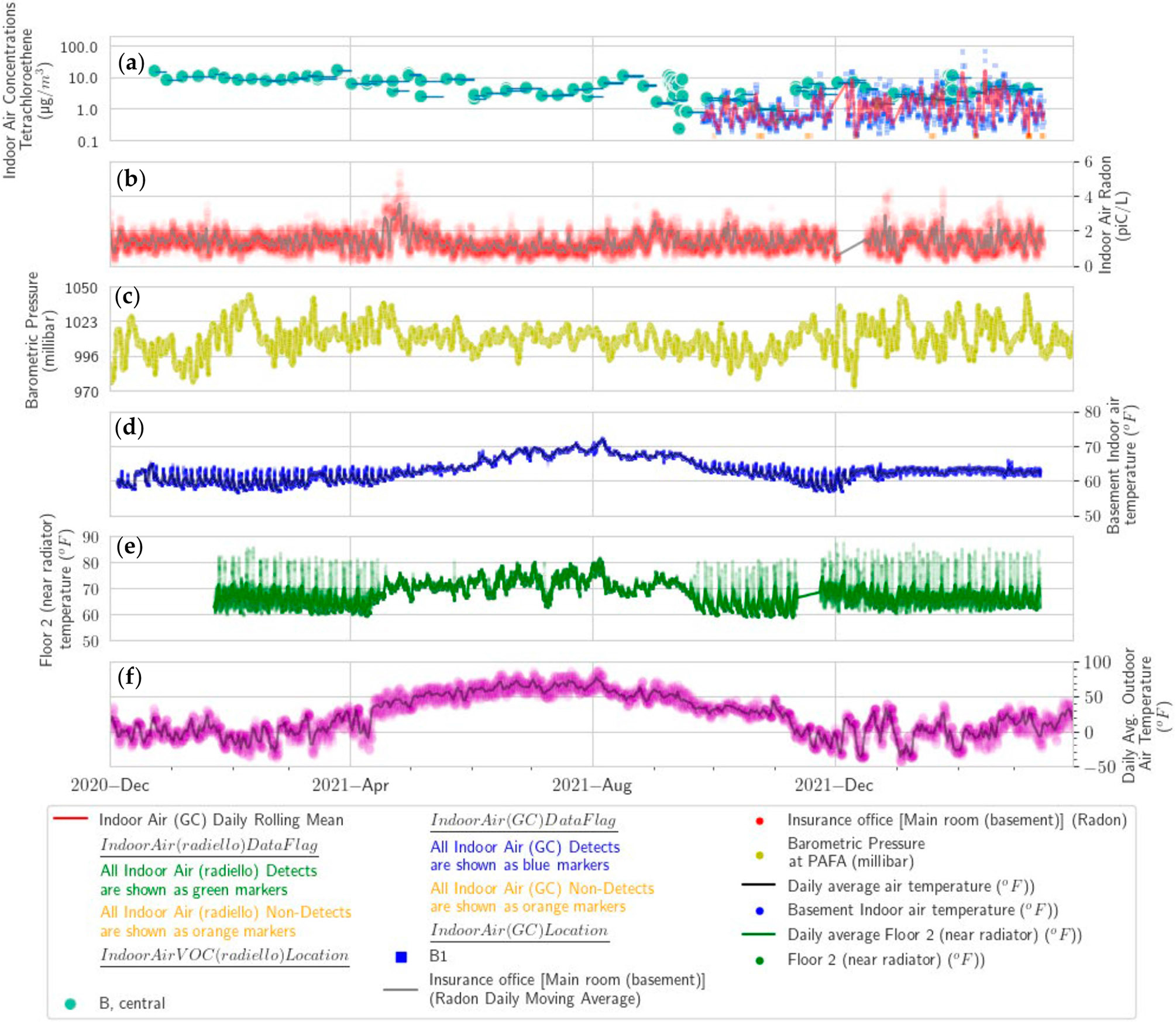
Residential-style office time series of: (**a**) Indoor air PCE Radiello^®^ (green circles), PCE GC (blue circles) and PCE GC daily rolling mean (red line), (**b**) radon (red circle) and daily moving average of radon (gray line), (**c**) barometric pressure (yellow circle), (**d**) basement temperature (blue circle), (**e**) second-floor temperature (green circle), and (**f**) outdoor temperatures (purple circle) and daily average air temperature (black line).

The residential-style office building is situated side-gradient or upgradient to the known sources and has been interpreted as being affected through a historic utility vapor corridor migration on the basis of the spatial patterns of subslab data and groundwater plume information [59]. A soil boring sample collected by another group in the immediate vicinity of this building [55] in November 2022 showed 56.6 μg/kg PCE at 0.9 m below ground surface (bgs; 3 ft bgs) and 63.8 μg/kg PCE at 2.9 m bls (9.5 ft bgs) in a sandy gravel matrix [60]. Calculations for a generic PCE source suggest that concentrations of 0.4 μg/kg in bulk soil are typically at equilibrium with a soil gas PCE concentration of 360 μg/m^3^, which confirms the presence of a substantial soil source near this building [61]. The highest PCE concentrations in indoor air and soil gas observed at this site were observed from January to May 2021 (Figure 9a). Generally, lower concentrations were observed in June, July, September, and October. A secondary peak was observed in August 2021. Increased concentrations in November and December2022 were observed but were confounded by higher exterior air concentrations, suggesting a possible regional or sitewide influence. Data from the GC/MS (Figure 10) suggests that PCE concentrations in outdoor air were sometimes equal to indoor air concentrations; however, the elevated concentrations in outdoor air were more persistent through this period. This suggests that vapor intrusion was occurring, but that under some wind or atmospheric conditions (such as local inversions), outgassing from the building or nearby soils was also a substantial source to the ambient monitoring location. However, the general correlation between PCE and radon peaks in indoor air suggests a soil gas intrusion influence (Figures 9b,c and 10). SSSG (Figure 9d) showed higher concentrations from January to March 2021 than from late August 2021 through January 2022, which suggests that soil temperature is not the dominant mechanism controlling indoor concentrations in this building.

A review of the overall time series for PCE and radon indoor air concentrations shows that several of the peaks in the radon time series, namely in May 2021, September 2021, and March 2022, are also reflected in the PCE time series (Figure 10). However, the radon peak in January 2021, although narrow, shows no clear equivalence in the weekly PCE time series. Moreover, the broad rise of PCE in November 2021 is not clearly reflected in the radon time series. This lack of agreement between the time series suggests that the vapor intrusion of PCE and radon is caused by a mix of some common and some disparate mechanisms. The greater degree of weekly scale variability in PCE indoor air concentrations starting in October 2021 and running into March 2022 appears to correlate with the greater degree of barometric pressure and exterior temperature variability during that period (Figure 10). The broad peak in the GC/ECD PCE data in November 2021 corresponds to the general/seasonal temperature reduction that occurred consistently during that month, presumably a result of the stack effect (Figure 10).

The low indoor concentrations in January and February and their rebound in March 2022 could potentially be attributable to a soil freezing depth effect since outdoor air temperatures went through a pronounced minimum in early February and began warming in late February through March. This interpretation is supported by a substantial decrease in one of the two subslab ports at this building in January/February. However, detailed soil temperature data for this date range and location are not available.

The church and the residential-style office buildings exhibited a common pattern with one prominent subslab (Figure 4) and indoor air peak (Figure 5) observed in late August/early September, consistent with the report of Barnes and McRae [26]. Barnes and McRae hypothesized that the pattern was due to the peak soil temperatures combined with the initiation of the winter heating season (and thus the stack effect). These buildings also have weeks with relatively high PCE from February to May, when soil temperatures are gradually warming (Figure S7). Broadly, the various sampling zones at any one building location track together in indoor air (Figure 5).

## 4. Conclusions

Indoor air VOC concentrations exhibited less variability at buildings near the VOC source (near the point of original release, RSD 43–51%) [55]. In contrast, the buildings more distant from the source exhibited a more variable seasonal distribution of indoor air VOC concentrations (RSD 47 to 95%) [55]. The dominant factors for the buildings not over the source area but over the groundwater plume are a peak in soil temperature, usually in late August or early September, combined with initiation of the building heating systems in the fall. The combination of these factors creates a stack effect within the buildings that increases the transport of contaminated soil vapor into them. At sites with high variability in soil permeability, soil temperature may be of limited use as an indicator. The dominant factor for buildings over the source (not-for-profit and tailor and tuxedo shop) is a seasonal summer high pattern with little to no effect from HVAC usage. The stack effect mechanisms, not associated with the effect of an increase in building air exchange due to HVAC systems during seasonal usage, had more influence on long-term temporal trends in VOC concentrations. Therefore, the HVAC systems and operations in the buildings across the site have little effect on long-term temporal trends in indoor air VOC concentrations other than by inducing the stack effect by warming the indoor air. With the clearer understanding of the relationship of I&Ts and their resulting effect upon the stack effect and vapor intrusion into buildings near and distant from the VOC source in this subarctic climatic zone, we can improve the timing of our assessment methods for VI in similar zones, resulting in the improvement of the protection of human health in indoor environments. Similar research studies within different climatic zones would provide validation of these findings.

## Supplementary Material

Supplement1

## Figures and Tables

**Table 1.** Building and HVAC characteristics.

Building	Building Characteristics					HVAC Characteristics						
	Description	Approximate m^2^ (ft^2^)	Number of Stories	General Con- struction Mate- rial/Style	Date of Con- struction, Additions and Major Renovations	Basement or Crawl Present?	Heating System	Heat Generation	Heat Distribution	Cooling System	Vent Systems	Number of Zones
**Eastern Plume/Source Area**												
Not for profit	Non-profit organization; Four-plex	369 (3975)	1	cinder block (property tax site says 8-inch concrete block)	1950	N	hot air circulation	boiler system appears to heat water to heat air; fuel oil used for boilers	air circulation ducts	central air (inlet appears to be from outside)	kitchen hood fan; bathroom fans	1
Tailor and tuxedo rental business	Tailor and tuxedo rentals; Four-plex	454 (4890)	1	cinder block	1950	N	hot water baseboard (electric)	boiler system; fuel oil for boiler	hot water to radiators at baseboards; space heater in storage rooms	central air (not used)	fans in storage rooms for in-room heating	1
Residential-style office	Residential-style building; business office	149 (1600)	2 and basement	wood frame over poured concrete	1951	Y, basement with separate access	hot water radiation. Baseboards	fuel oil for heating	hot water to radiators at baseboards likely		bathroom vents	3
Government building	Large government building with basement	6500 (70,000)	2 and basement	primarily reinforced concrete	Constructed in 1933-34 with additions in 1939 and 1948. Renovations in 1994.	Y, basement partially below grade	hot water radiation	district steam plant	radiators in most offices; air handling room on 3rd floor includes heated coils (steam)	Central air in some areas; most offices have individual AC units	central ventilation with large vents in most portions of the building	
**Western Plume/Source Area**												
Office Building	first floor office	156 (1680)	2	concrete slab; brick and wood frame	1970	N	hot water radiation	fuel oil	hot water to radiators at baseboards likely	1st floor: central air, outside intake; 2nd floor: individual AC units,	1st floor: fans, bathroom fans; 2nd floor: fans, bathroom fans	3
	second floor office											
Church	Place of worship	557 (6000)	split level first floor over basement	cinder block walls in basement; wood frame above	1952	Y, basement partially below grade	plate exchanger with hot water radiation (baseboards)	hot water from city central hot water; fuel oil	hot water to radiators at baseboards likely		bathroom vents, outside air intake with exhaust at south end of chapel	Possibly 6

**Table 2.** PCE summary statistics of detections and calculated attenuation factors.

Building	Indoor Air Loca- tion/Sample ID	Long Term Average Indoor Air PCE ^1^	Standard Deviation Indoor Air PCE	Subslab Loca- tion/Sample ID	Long Term Average Subslab PCE	Standard Deviation Subslab PCE	SS AF, Corrected for Ambient (Unitless)	SS AF Standard Deviation
Church	Basement (Main Room, South end); IA-50	11.23 (*n* = 57)	5.33	Basement (Main Room, Central; Storage Room, SE) SS-54, SS-56	427.28 (*n* = 152)	363.33	0.0202	0.0196
Church	Basement (Kitchen); IA-51	12.03 (*n* = 59)	7.43	Basement (Storage Room, NW); SS-55	247.49 (*n* = 82)	154.47	0.0381	0.0334
Residential-style Office	Basement; IA-43	5.49 (*n* = 108)	3.63	Basement (Central Closet; East, bottom of stairs) SS-59, SS-60	621.31 (*n* = 159)	383.32	0.0046	0.0042
Tailor and Tuxedo	Tuxedo room; IA-44	33.74 (*n* = 58)	15.22	Front desk, N; SS-48	942.17 (*n* = 63)	413.61	0.0330	0.0208
Tailor and Tuxedo	Central half-wall; IA-45	29.05 (*n* = 56)	14.85	Storage Room, S; SS-49	11957.66 (*n* = 64)	9127.20	0.0022	0.0020
Not For Profit	Hallway near kitchen; IA-47	13.57 (*n* = 58)	5.93	Mech Room, SE; SS-53	3407.58 (*n* = 62)	2064.16	0.0032	0.0024
Not For Profit	Conference room; IA-46	12.16 (*n* = 58)	5.28	Conference Room NW; SS-51	2490.24 (*n* = 57)	1299.33	0.0038	0.0026
Office building	First floor; IA-48	3.5 (*n* = 58)	2.18	East office 2; SS-57	255.14 (*n* = 14)	677.90	0.0035	0.0095
Outdoor Air		2.61 (*n* = 86)	2.75	NA	NA	NA	NA	NA

Notes: ^1^ Long-term average indoor air is defined as those with sample durations greater than 4 days. *n* = number of samples, NA = Not Applicable.

## Data Availability

The original data presented in the study are openly available at DATA.gov or DOI: 10.23719/1529842.
