# Supplementary material for: Impact of Multiple HVAC Systems on Indoor Air VOC and Radon Concentrations from Vapor Intrusion During Seasonal Usage
Source: Atmosphere (Basel). Author manuscript; Available in PMC 2026 Apr 1. (PMC12180761; doi:10.3390/atmos16040378)
Supplement: Supplement1 [file NIHMS2071798-supplement-Supplement1.pdf]

Impact of Multiple HVAC Systems on Indoor Air VOC and Radon Concentrations from Vapor Intrusion During Seasonal Usage

John H. Zimmerman[a*], Alan Williams[a], Brian Schumacher[b], Chris Lutes[c], Rohit Warrier[e], Brian Cosky[c], Ben Thompson[c], Chase Holton[d], and Kate Bronstein[e]

[a.] U.S. Environmental Protection Agency, 109 TW Alexander Drive, P.O. Box 12055, RTP, NC 27711
[b.] U.S. EPA, 960 College Station Road, Athens, GA 30605
[c.] Jacobs, 1999 Bryan St., Suite. 1200, Dallas, TX 75201
[d.] GSI Environmental Inc., 13949 W. Colfax, No. 210, Lakewood, CO 80401
[e.] RTI International, 3040 E. Cornwallis Rd., Research Triangle Park, NC 27709
**Corresponding author: zimmerman.johnh@epa.gov*

Supplemental Information

Table S1. Summary of Sampling Locations by Building

Building	Sample Type	Number of Sample locations	Sample location within building	Sample ID	COC	Operational Period (Month, Date, Year)
**Eastern Plume/Source Area**						
Not-for-Profit	Indoor Air	2	NW conference room	IA-46	VOC	12/23/2020 to 3/20/2022
			hallway adjacent to kitchen	IA-47	VOC	12/23/2020 to 3/20/2022
	Subslab	3	conference room, NW	SS-51	VOC and Radon	12/1/2020 to 3/20/2022
			mech room, SE	SS-53	VOC and Radon	12/1/2020 to 3/20/2022
			main central dining room	SS-52	VOC and Radon	10/23/2020 (radon), 12/1/2020 (VOC) to 3/20/2022
	Diff. Press.	2	near mech room, SE	NA	Differential pressure, Temperature	12/7/2020 to 3/14/2022
			main central dining room	NA	Differential pressure, Temperature	12/23/2020 to 3/14/2022
Tailor and tuxedo rental business	Indoor Air	2	storeroom, central halfwall	IA-44	VOC	12/22/2020 to 3/20/2022
			storage room west, tuxedo room	IA-45	VOC	12/22/2020 to 3/20/2022
	Subslab	3	front desk, N	SS-48	VOC and Radon	12/1/2020 to 3/20/2022
			storage room, S	SS-49	VOC and Radon	12/1/2020 to 3/20/2022
			central closet	SS-50	VOC and Radon	12/9/2020 to 3/20/2022
	Groundwater	2	south of building	MW31	VOC	4/1/2021 (first quarter)
			north of building	MW33s	VOC	4/1/2021 (first quarter)
	Radon	2	main front room	NA	Radon	10/23/2020 to 3/20/2022
			west storage	NA	Radon	12/1/2020 to 3/20/2022
	Diff. Press.	2	main front room	NA	Differential pressure, Temperature	12/7/2020 to 3/15/2022
			by back dock	NA	Differential pressure, Temperature	12/7/2020 to 3/15/2022
	Temp.	1	front entry room	NA	Indoor air temperature	1/22/2021 to 3/15/2022
Residential-style office	Indoor Air	2	Floor 1, kitchen/office	IA-42	VOC	12/23/2020 to 3/20/2022
			Basement, central	IA-43	VOC	12/23/2020 to 3/20/2022
	Indoor Air (GC)	5	Basement 1	IA-63	VOC	9/25/2021 to 3/16/2022
			Basement 2	IA-65	VOC	9/25/2021 to 3/16/2022
			Garage	IA-66	VOC	9/29/2021 to 2/18/2022
			Floor 1, office	IA-67	VOC	9/25/2021 to 3/16/2022
			Floor 1, kitchen	IA-68	VOC	9/25/2021 to 3/16/2022
	Outdoor Air (GC)	2	OA-14 was relocated to OA-15 on 10/1/2021	OA-14	VOC and Radon	9/25/2021 to 10/1/2021
			OA-14 was relocated to OA-15 on 10/1/2021	OA-15	VOC and Radon	10/1/2020 to 3/16/2022
	Subslab (GC)	2	Basement, central closet	SS-59	VOC and Radon	12/9/2020 to 3/20/2022
			Basement, east, bottom of stairs	SS-60	VOC and Radon	12/9/2020 to 3/20/2022
	Radon	2	Floor 1, kitchen/office	NA	Radon	12/9/2020 to 3/20/2022
			Basement, main room	NA	Radon	10/23/2020 to 3/20/2022
	Diff. Press.	2	Basement, main room	NA	Differential pressure, Temperature	12/7/2020 to 3/15/2022
			Former garage	NA	Differential pressure, Temperature	12/4/2020 to 3/15/2022
	Temp.	1	Floor 1, kitchen/office	NA	Indoor air temperature	1/22/2021 to 3/15/2022

Building	Sample Type	Number of Sample locations	Sample location within building	Sample ID	COC	Operational Period (Month, Date, Year)
Government building	Indoor Air	7	Basement, B&G club teen center, kitchen counter1	IA-35	VOC	12/22/2020 to 3/20/2022
			Basement, SW corner file room1	IA-36	VOC	12/22/2020 to 3/20/2022
			Basement, SE corner storage room1	IA-38	VOC	12/22/2020 to 3/20/2022
			Basement, NE corner, maintenance crew shop	IA-39	VOC	12/23/2020 to 3/20/2022
			Floor 1, B&G club gym, front desk	IA-36	VOC	12/22/2020 to 3/20/2022
			Floor 1, SW corner engineering technicians' office equipment area	IA-40	VOC	12/22/2020 to 3/20/2022
			Floor 1, SE corner engineering office	IA-41	VOC	12/22/2020 to 3/20/2022
	Subslab	3	B, gym, central	SS-45	VOC and Radon	12/1/2020 to 3/20/2022
			B, SW file room	SS-47	VOC and Radon	12/1/2020 to 3/20/2022
			B, B&G club teen center, NW	SS-46	VOC and Radon	12/1/2020 to 3/20/2022
	Radon	4	Basement, B&G club teen center, kitchen counter	NA	Radon	12/1/2020 to 3/20/2022
			Basement, SW corner file room	NA	Radon	10/22/2020 to 3/20/2022
			Floor 1, B&G club gym, front desk	NA	Radon	12/1/2020 to 3/20/2022
			Floor 1, SE corner engineering office	NA	Radon	12/3/2020 to 3/20/2022
	Diff. Press.	2	Basement, B&G club teen center, kitchen	NA	Differential pressure, Temperature	12/7/2020 to 3/15/2022
			in southeast stairwell from basement to floor 1	NA	Differential pressure, Temperature	12/4/2020 to 3/15/2022
**Western Plume/Source Area**						
Office Building	Indoor Air	1	F1, central reception on cabinet	IA-48	VOC	12/16/2020 to 3/8/2022
	Subslab	2	F1, east office 2	SS-57	VOC and Radon	12/10/2020 to 3/14/2022
			F1, mech room	SS-58	VOC and Radon	12/10/2020 to 3/14/2022
	Radon		F1, main entrance	NA	Radon	10/23/2020 to 3/14/2022
	Diff. Press.	2	F1, boiler room	NA	Differential pressure, Temperature	12/7/2020 to 3/14/2022
			stairwell floors 1 and 2	NA	Differential pressure, Temperature	12/15/2020 to 3/14/2022
	Indoor Air	1	F2, cabinet	IA-49	VOC	12/16/2020 to 3/8/2022
	Radon	1	F2, main entrance	NA	Radon	12/2/2020 to 3/14/2022
	Temp.	1	F2 reception	NA	Indoor air temperature	1/22/2021 to 3/14/2022
Church	Outdoor Air	1	south end, away from building	OA-12	VOC	12/23/2020 to present
	Outdoor Air (GC)	1	Outside mechanical room vent	OA-13	VOC	1/25/2022
	Indoor Air	3	B, main room, south end1	IA-50	VOC	12/16/2020 to 3/8/2022
			B, kitchen1	IA-51	VOC	12/16/2020 to 3/8/2022
			F2, chapel1	IA-52	VOC	12/16/2020 to 3/8/2022
	Indoor Air (GC)	8	Mechanical room	IA-57	VOC	8/26/2021 to 2/9/2022
			Mechanical room (moved from IA-57 on 2/9/2022 to opposite wall)	IA-69	VOC	2/9/2022 to 3/18/2022
			SE corner	IA-58	VOC	8/26/2021 to 3/18/2022
			Classroom	IA-59	VOC	8/26/2021 to 3/18/2022
			Entryway	IA-60	VOC	8/26/2021 to 3/18/2022
			Chapel corner	IA-61	VOC	8/26/2021 to 3/18/2022
			Chapel mid	IA-62	VOC	8/26/2021 to 3/18/2022
			Main room, south end	IA-50	VOC	8/26/2021 to 3/18/2022
	Subslab (GC)	3	F1, main room, central	SS-54	VOC and Radon	12/1/2020 to 3/14/2022
			F1, storage room, NW	SS-55	VOC and Radon	12/1/2020 to 3/14/2022
			F1, storage room, SE	SS-56	VOC and Radon	12/9/2020 to 3/14/2022
	Soil Vapor	16	west, 2'	SG-16	VOC and Radon	12/10/2020 to 3/16/2022
			west, 4.5' (GC location)	SG-17	VOC and Radon	12/10/2020 to 3/16/2022
			west, 7' (GC location)	SG-18	VOC and Radon	12/10/2020 to 3/16/2022
			west, 9.5'	SG-19	VOC and Radon	12/10/2020 to 3/16/2022
			east, 2'	SG-20	VOC and Radon	12/10/2020 to 3/16/2022
			east, 4.5'	SG-21	VOC and Radon	12/10/2020 to 3/16/2022

Building	Sample Type	Number of Sample locations	Sample location within building	Sample ID	COC	Operational Period (Month, Date, Year)
			east, 7'	SG-22	VOC and Radon	12/10/2020 to 3/16/2022
			east, 9.5'	SG-23	VOC and Radon	12/10/2020 to 3/16/2022
			south, 2'	SG-24	VOC and Radon	12/10/2020 to 3/16/2022
			south, 4.5' (GC location)	SG-25	VOC and Radon	12/10/2020 to 3/16/2022
			south, 7' (GC location)	SG-26	VOC and Radon	12/10/2020 to 3/16/2022
			south, 9.5'	SG-27	VOC and Radon	12/10/2020 to 3/16/2022
			north, 2'	SG-28	VOC and Radon	12/10/2020 to 3/16/2022
			north, 4.5'	SG-29	VOC and Radon	12/10/2020 to 3/16/2022
			north, 7'	SG-30	VOC and Radon	12/10/2020 to 3/16/2022
			north, 9.5'	SG-31	VOC and Radon	12/10/2020 to 3/16/2022
	Diff. Press.	3	Kitchen (basement to floor 1)	NA	Differential pressure, Temperature	12/4/2020 to 3/14/2022
			mech room (indoor to outdoor)	NA	Differential pressure, Temperature	12/8/2020 to 11/17/2021<br>12/8/2021 to 3/14/2021
			central meeting room (SS to indoor)	NA	Differential pressure, Temperature	12/7/2020 to 11/24/2021<br>1/5/2022 to 1/21/2022<br>2/9/2022 to 3/14/2022
	Temp.	1	auditorium	NA	Indoor air temperature	1/22/2021 to 3/15/2022
	Radon	3	auditorium	NA	Radon	11/5/2020 to present
			central meeting room	NA	Radon	10/22/2020 to present
			ambient by Rad7	NA	Radon	12/8/2020 to present

Sources and Conceptual Site Models

Sources and conceptual site models—eastern plume

The eastern plume is thought to originate from a laundromat that has been operational since at least 1960 and did dry cleaning on-site until 1998, when it transitioned to providing drop-off services for off-site dry-cleaning work and on-site coin-operated laundry operations [62]. This dry cleaner in a "four-plex" of abutting or nearly abutting buildings separated by alleys facing Gaffney Road (Figure S1). The four-plex consists of the (a) dry cleaner, (b) a Not-for-Profit organization, (c) a tuxedo and tailor shop, and (d) a nonprofit office/retailer.

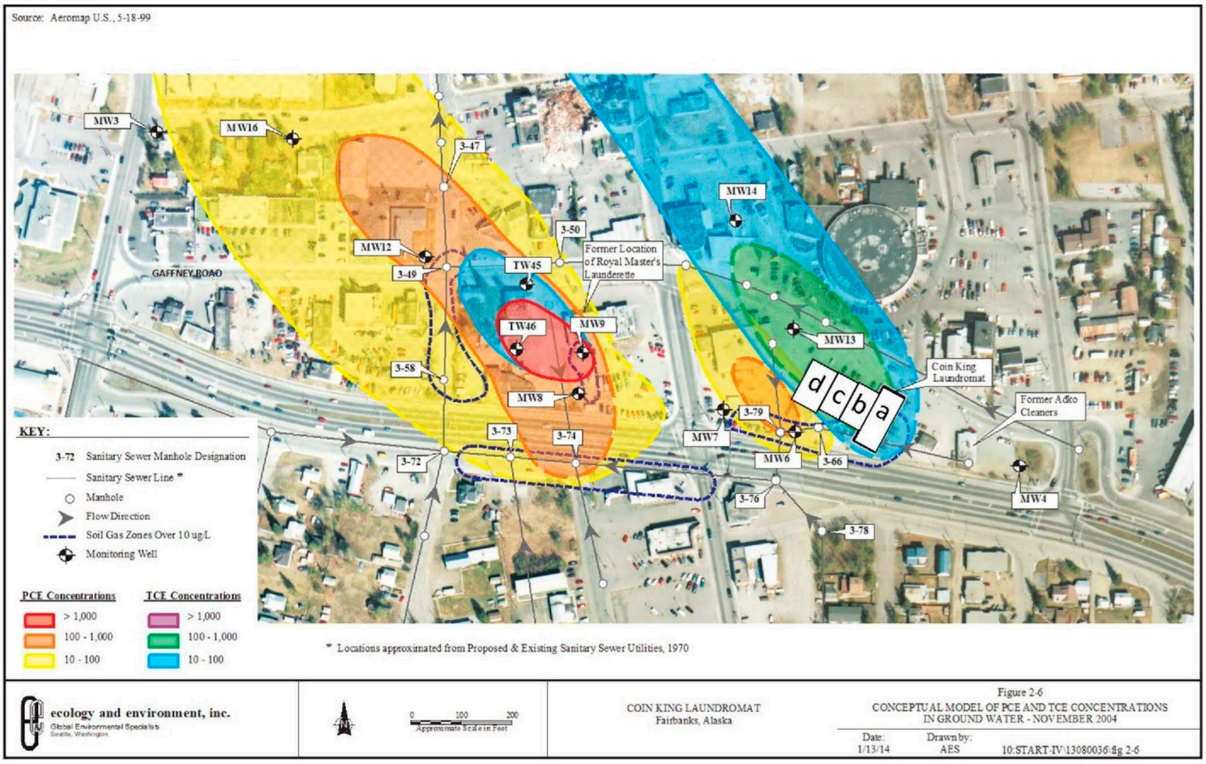

Figure S1. Eastern and western plumes close to their sources, Fairbanks, Alaska [36].

Buildings (b) and (c) were monitored along with four other structures (discussed below) in this study. Several multimedia investigations have been performed to characterize the source, nature, and extent of release (e.g., reports from Oasis Environmental [36, 59, 63]. A second dry cleaner lies approximately 45.7 m (150 feet [ft]) to the east.

In general, the highest soil contaminant concentrations identified were near the southeast corner of the former dry cleaner building, where the historic presence of a drum storage area was noted [37]. Tetrachloroethylene (PCE) was detected in soil samples collected between 0 and 4.6 m (0 and 15 ft) below ground surface, with a maximum PCE concentration of 82,000 micrograms per kilogram (µg/Kg) collected from boreholes on the south side of the dry cleaner building. The soil source area extends from the back of the building out under the pavement on Airport Way [37]. As in the soil, elevated PCE concentrations in groundwater have been reported near the former drum storage area south of the dry cleaner building, with a maximum PCE concentration of 4,100 micrograms per liter (µg/L). As of 2014, a detectable groundwater solvent plume extends from this area northwest to 10th Avenue (Figure S1). Statistical analyses suggest that the groundwater plume is stable [36].

Vapor intrusion (VI) assessments have been conducted at the four-plex and the grocery store/market, located downgradient of the dry cleaner. These assessments included collection and analysis of subslab soil gas (SSSG), indoor air, and outdoor air samples. Table S2 summarizes VI samples historically reported at each building. Indoor air samples collected from the ground/first floor and the basement at the dry cleaner building detected several volatile organic compounds (VOCs) at concentrations higher than those of outdoor ambient air. Notably, PCE concentrations ranged from 15 to 20 micrograms per cubic meter (µg/m^3), whereas trichloroethylene (TCE) concentrations ranged from 1.3 to 7.0 µg/m^3 and were below Alaska Department of Environmental Conservation (ADEC) target levels for commercial indoor air at the time of sampling (PCE at 180 µg/m^3; TCE at 8.8 µg/m^3). These indoor air VOCs were not definitively attributed to a subsurface source because of the presence of freshly dry-cleaned clothes in the building at the time of sampling and the absence of concurrent SSSG samples. Therefore, the presence of a complete VI pathway at the dry cleaner building was not concluded [37]. However, the VI pathway was determined to be complete at the other three buildings within the four-plex. SSSG and indoor air samples were collected from these buildings and reported detectable PCE and TCE

concentrations. PCE concentrations in SSSG ranged from 280 µg/m^3 to 44,000 µg/m^3 and exceeded the ADEC target level of 210 µg/m^3 at each point sampled. Of the three buildings sampled, indoor air PCE and TCE concentrations only exceeded ADEC target levels at the tuxedo and tailor shop. These exceedances may have resulted from the large quantity and variety of cleaning products and other chemicals stored and the small amount of dry-cleaned clothes present during the sampling event [36].

Table S2. Historical VOC Sampling Summary

Business	Overlying Eastern/ Western Plume	Time Period	Sampling Events (#)	Events per Season (#)	Types and Number of Samples	Max PCE Concentration (µg/m³)	Median PCE Concentration (µg/m³)	Max TCE Concentration (µg/m³)	Median TCE Concentration (µg/m³)
Dry-cleaner	Eastern	2013	1	Winter: 0	Subslab: 0	--	--	--	--
				Spring: 0	Indoor: 3	20	20	7	4.3
				Summer: 0	Long-term passive: 0	--	--	--	--
				Fall: 1	Crawl space: 0	--	--	--	--
					Exterior soil gas: 2	ND (0.23)	ND (0.21)	ND (0.18)	ND (0.16)
Residential-style Office	Eastern	2008–2011	9	Winter: 3	Subslab: 18	19,000	7,300	860	335
				Spring: 2	Indoor: 11	4.3	1.7	0.19	ND (0.18)
				Summer: 1	Long-term passive: 0	--	--	--	--
				Fall: 3	Crawl space: 0	--	--	--	--
					Exterior soil gas: 2	5,900	5,150	400	355
Private residence	Western	2002–2013	6	Winter: 3	Subslab: 7	19,000	1,500	56	11
				Spring: 0	Indoor: 9	51	33	1	ND (4.6)
				Summer: 1	Long-term passive: 0	--	--	--	--
				Fall: 2	Crawl space: 0	--	--	--	--
					Exterior soil gas: 0	--	--	--	--
Church	Western	2007	1	Winter: 0	Subslab: 3	1,700	34	ND (2.6)	ND (2.6)
				Spring: 0	Indoor: 1	0.68	0.68	ND (0.14)	ND (0.14)
				Summer: 1	Long-term passive: 0	--	--	--	--
				Fall: 0	Crawl space: 0	--	--	--	--
					Exterior soil gas: 0	--	--	--	--
Residence	Western	2007	1	Winter: 0	Subslab: 3	310	120	16	3.6
				Spring: 0	Indoor: 1	0.25	0.25	ND (0.14)	ND (0.14)

Business	Overlying Eastern/ Western Plume	Time Period	Sampling Events (#)	Events per Season (#)	Types and Number of Samples	Max PCE Concentration (µg/m³)	Median PCE Concentration (µg/m³)	Max TCE Concentration (µg/m³)	Median TCE Concentration (µg/m³)
				Summer: 1	Long-term passive: 0	--	--	--	--
				Fall: 0	Crawl space: 0	--	--	--	--
					Exterior soil gas: 0	--	--	--	--
Restaurant/bar	Eastern	2008	1	Winter: 0	Subslab: 0	--	--	--	--
				Spring: 0	Indoor: 0	--	--	--	--
				Summer: 0	Long-term passive: 0	--	--	--	--
				Fall: 1	Crawl space: 0	--	--	--	--
					Exterior soil gas: 4	620	228	ND (9.1)	ND (7.6)
Non-profit organization (literary)	Eastern	2009–2014	7	Winter: 2	Subslab: 20	40,000	1,150	1,200	12
				Spring: 2	Indoor: 13	47	9.8	0.68	0.4
				Summer: 1	Long-term passive: 2	6.0	5.55	0.12	0.115
				Fall: 2	Crawl space: 0	--	--	--	--
					Exterior soil gas: 2	9,500	5,700	150	ND (75)
Tailor and tuxedo rental business	Eastern	2009–2011	6	Winter: 2	Subslab: 14	44,000	8,950	1,500	200
				Spring: 1	Indoor: 7	8,500	68	140	2
				Summer: 1	Long-term passive: 0	--	--	--	--
				Fall: 2	Crawl space: 0	--	--	--	--
					Exterior soil gas: 4	29,000	11,100	2,000	260
Not-for-Profit	Eastern	2009–2014	7	Winter: 2	Subslab: 14	17,000	970	85	9.45
				Spring: 1	Indoor: 8	41	18.5	0.61	0.53
				Summer: 2	Long-term passive: 2	11	10.05	0.16	0.135
				Fall: 2	Crawl space: 0	--	--	--	--
					Exterior soil gas: 2	1,600	1,055	64	ND (32)

Business	Overlying Eastern/ Western Plume	Time Period	Sampling Events (#)	Events per Season (#)	Types and Number of Samples	Max PCE Concentration (μg/m³)	Median PCE Concentration (μg/m³)	Max TCE Concentration (μg/m³)	Median TCE Concentration (μg/m³)
Grocery store/ market	Eastern	2011	2	Winter: 1	Subslab: 8	1,100	24	8.6	4.7
				Spring: 0	Indoor: 6	3.9	3.6	0.13	0.072
				Summer: 0	Long-term passive: 0	--	--	--	--
				Fall: 1	Crawl space: 0	--	--	--	--
					Exterior soil gas: 0	--	--	--	--
Non-profit organization (fourplex)	Eastern	2011	2	Winter: 1	Subslab: 2	530	520	300	285
				Spring: 0	Indoor: 2	1.2	1.35	0.081	0.076
				Summer: 0	Long-term passive: 0	--	--	--	--
				Fall: 1	Crawl space: 0	--	--	--	--
					Exterior soil gas: 0	--	--	--	--
Fire station	Eastern	2014	1	Winter: 0	Subslab: 0	--	--	--	--
				Spring: 1	Indoor: 0	--	--	--	--
				Summer: 0	Long-term passive: 1	0.14	0.14	ND (0.049)	ND (0.049)
				Fall: 0	Crawl space: 0	--	--	--	--
					Exterior soil gas: 0	--	--	--	--
Office Building	Western	2002–2007	5	Winter: 3	Subslab: 6	ND (6.5)	ND (5.2)	ND (5.1)	ND (4.2)
				Spring: 0	Indoor: 8	6	3.2	0.67	0.38
				Summer: 1	Long-term passive: 0	--	--	--	--
				Fall: 1	Crawl space: 0	--	--	--	--
					Exterior soil gas: 0	--	--	--	--

A nearby residential-style structure used as an office was believed to have been affected by the eastern plume through a primarily sewer transport mechanism and was included as part of this study (Figure 6). This building has a basement approximately 6 feet deep [59], an attached former garage, and two above-ground stories. The building is a bungalow- or cape-style construction [67] and was built in 1951 with a wood frame [68]. This building was sampled between 2008 and 2011, with maximum subslab concentrations of 19,000 µg/m^3 (PCE) and 860 µg/m^3 (TCE); maximum indoor air concentrations were 4.3 µg/m^3 (PCE) and 0.19 µg/m^3 (TCE) (Table S2 [14]). The VI pathway for this building was determined to be complete, although PCE concentrations in the workspace at the time of sampling were below ADEC target levels [64].

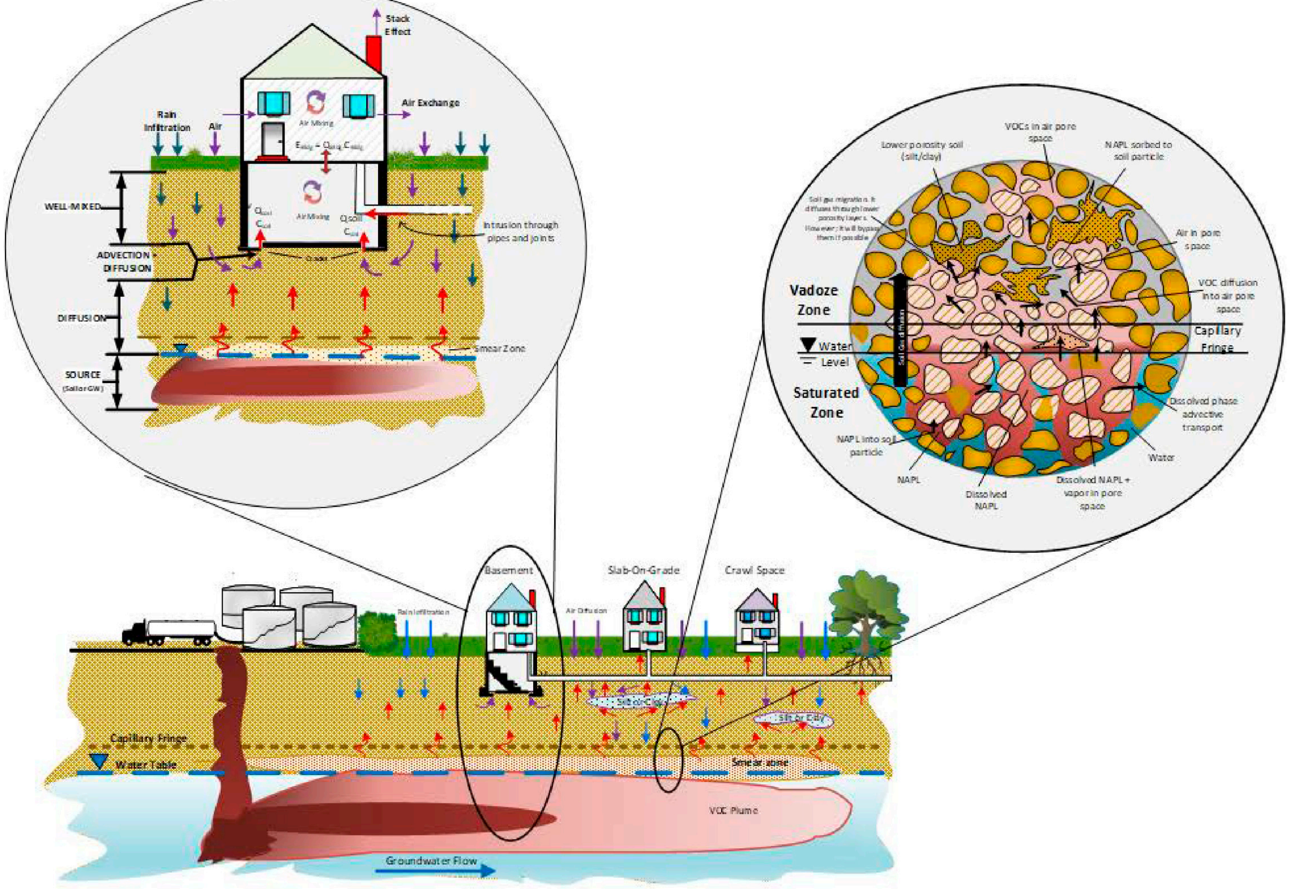

Figure S2. Vapor intrusion conceptual site model diagram (used by permission, Jacobs Engineering).

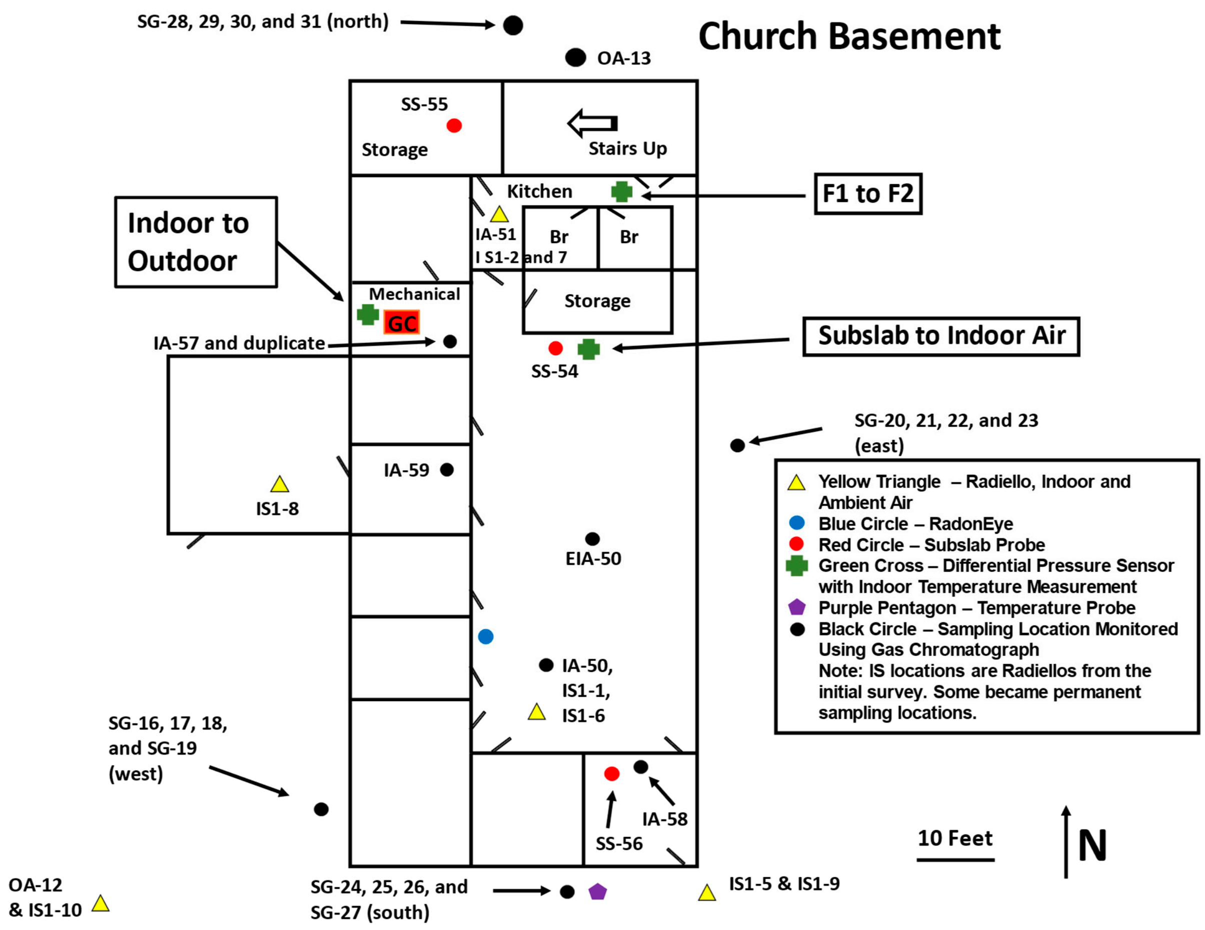

Figure S3a. Church sampling locations.

Church 2nd Floor

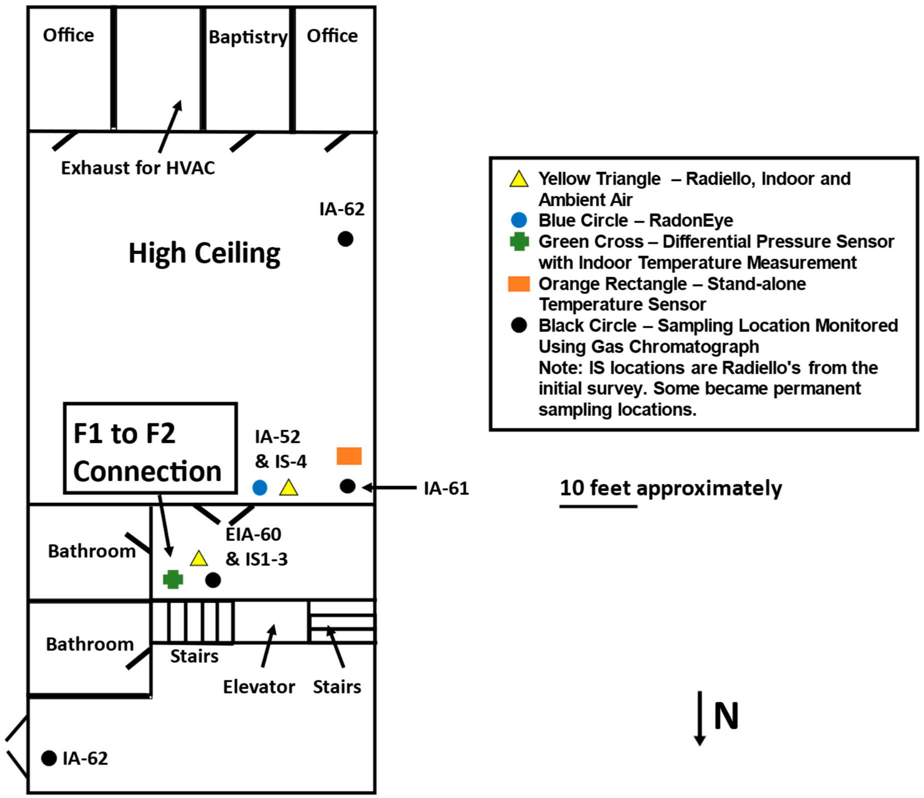

Figure S3b. Church second floor.

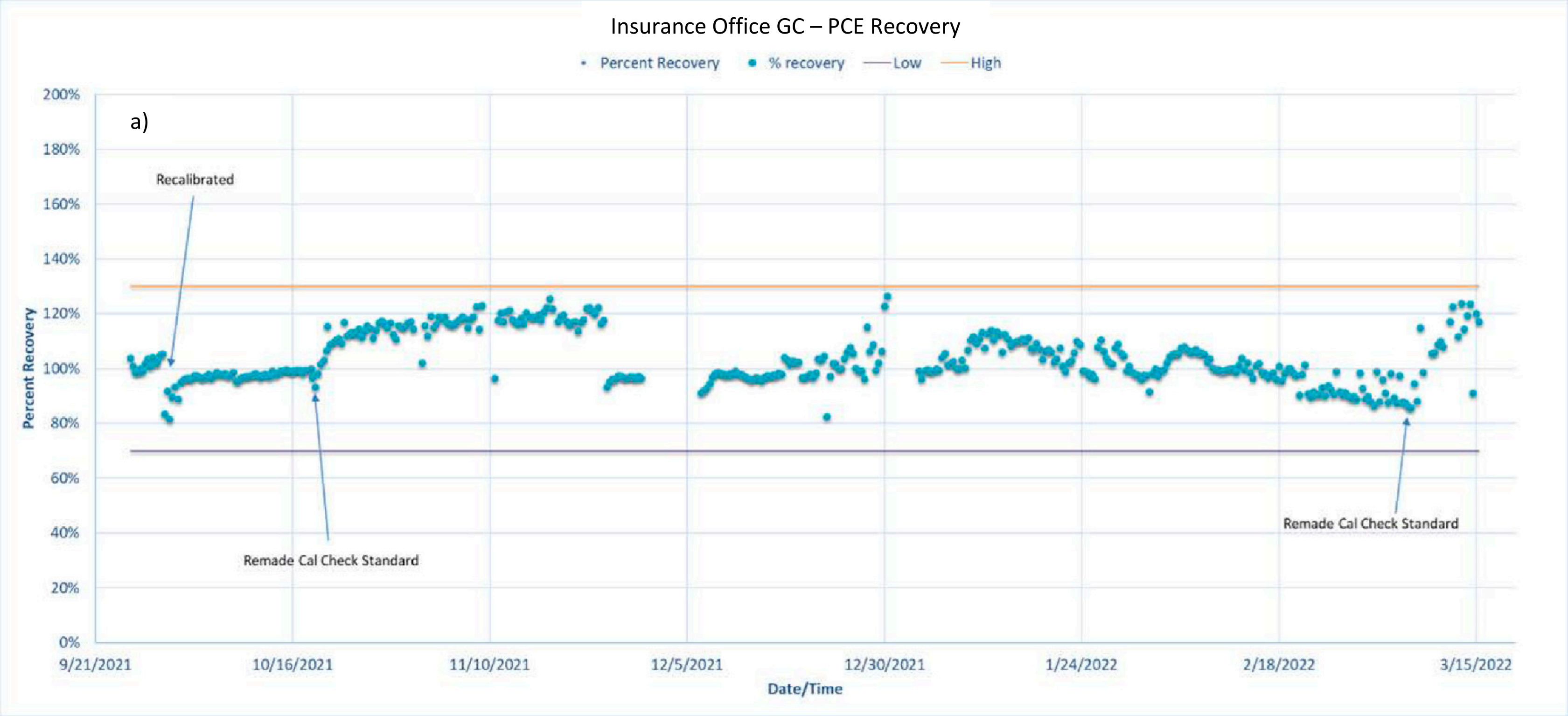

Insurance Office GC – PCE Recovery
Percent Recovery
% recovery
Low
High
a)
Recalibrated
Remade Cal Check Standard
Remade Cal Check Standard
Percent Recovery
200%
180%
160%
140%
120%
100%
80%
60%
40%
20%
0%
9/21/2021
10/16/2021
11/10/2021
12/5/2021
12/30/2021
1/24/2022
2/18/2022
3/15/2022
Date/Time

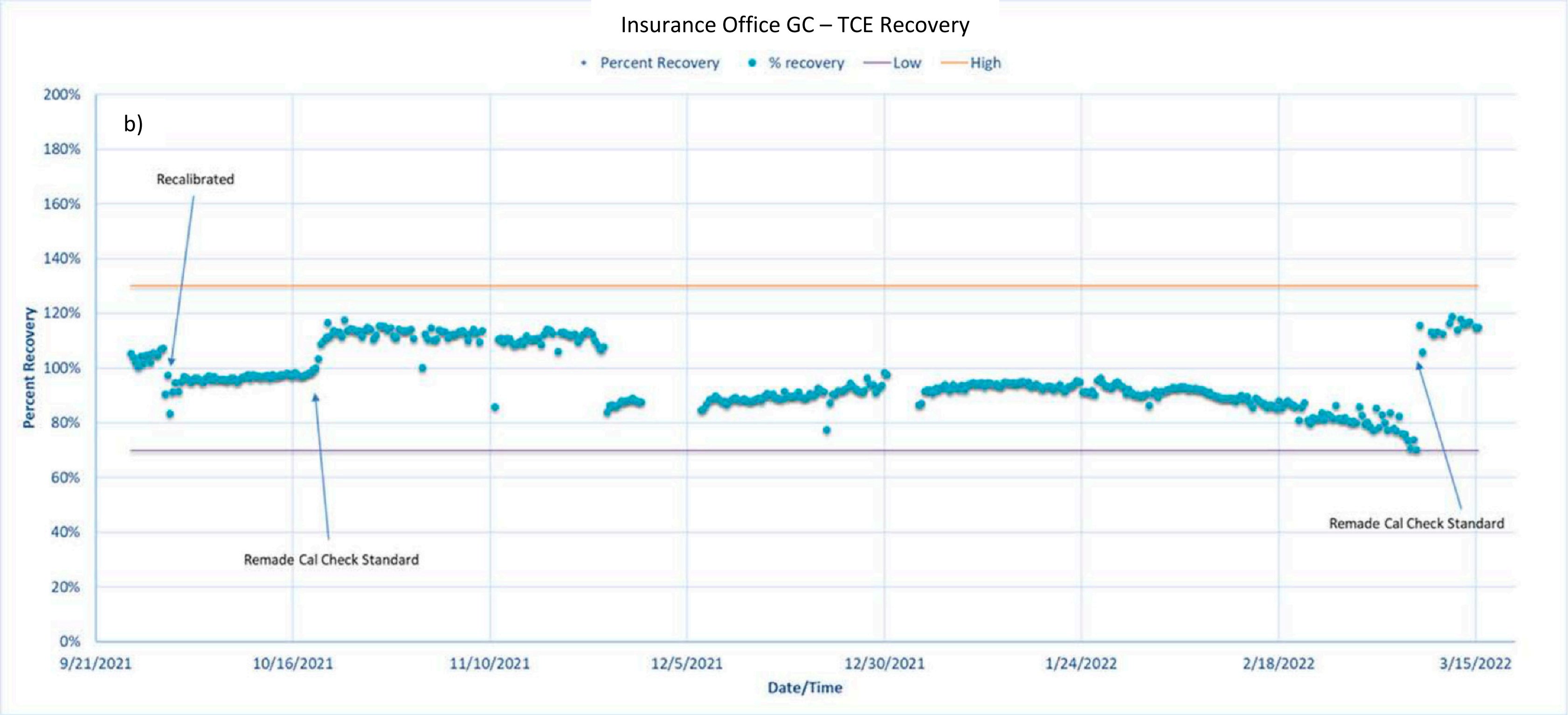
Insurance Office GC – TCE Recovery
Percent Recovery
% recovery
Low
High
b)
Recalibrated
Remade Cal Check Standard
Remade Cal Check Standard
Percent Recovery
200%
180%
160%
140%
120%
100%
80%
60%
40%
20%
0%
9/21/2021
10/16/2021
11/10/2021
12/5/2021
12/30/2021
1/24/2022
2/18/2022
3/15/2022
Date/Time

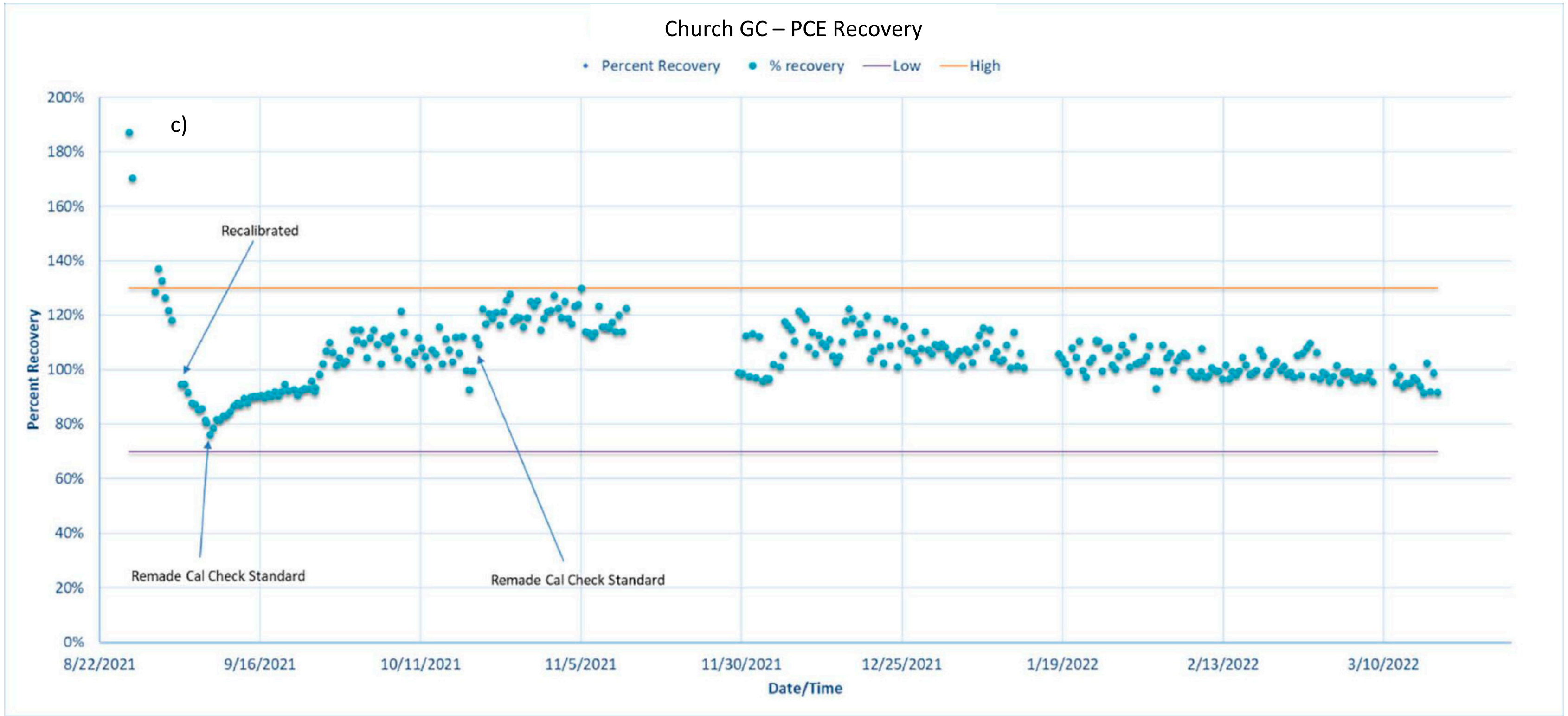
Church GC – PCE Recovery
Percent Recovery
% recovery
Low
High
c)
200%
180%
160%
140%
120%
100%
80%
60%
40%
20%
0%
Percent Recovery
Recalibrated
Remade Cal Check Standard
Remade Cal Check Standard
8/22/2021
9/16/2021
10/11/2021
11/5/2021
11/30/2021
12/25/2021
1/19/2022
2/13/2022
3/10/2022
Date/Time

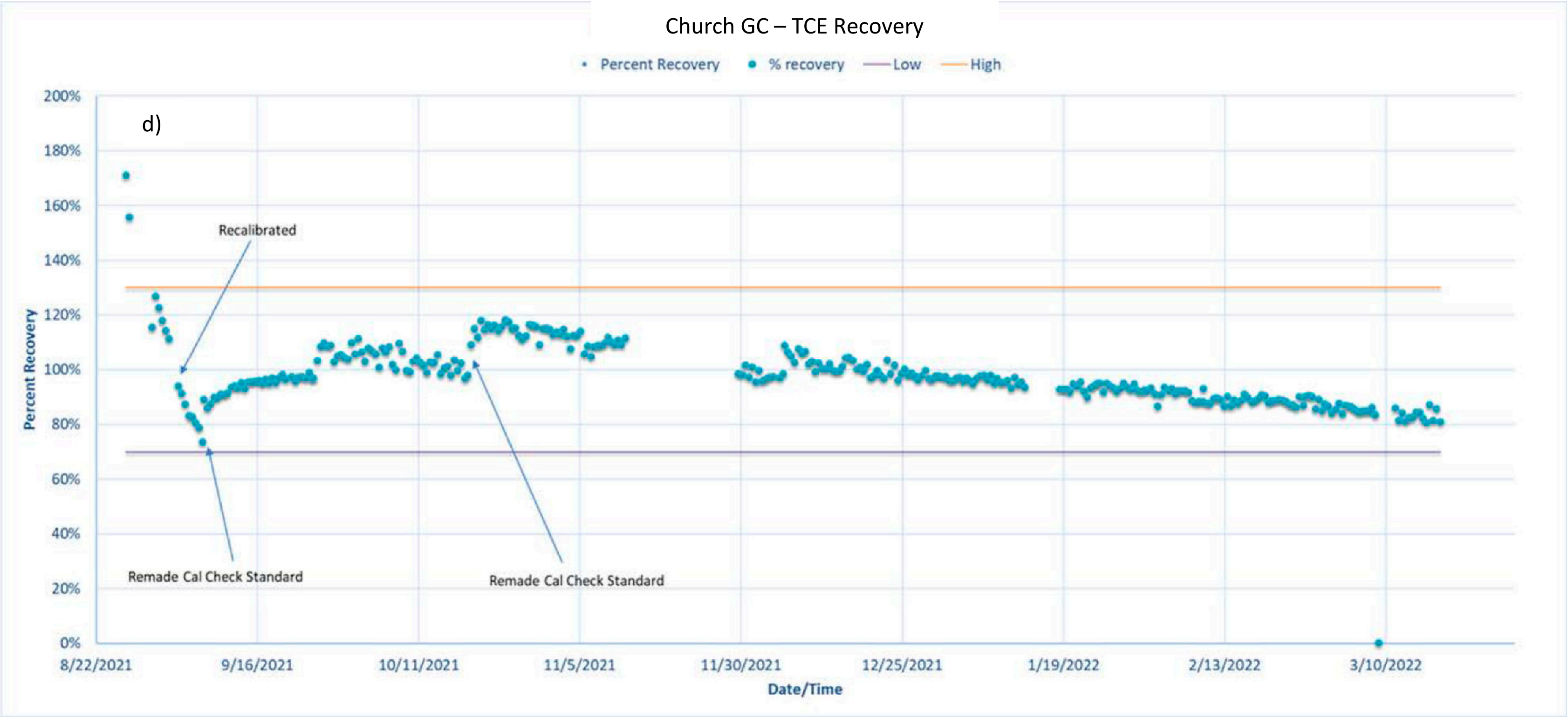

Figure S4. Continuous VOC data were collected at the church and insurance office using two separate GC/ECD instruments: a) Insurance Office-PCE, b) Insurance Office-TCE, c) Church-PCE, d) Church-TCE.

Sources and CSM—western plume

The western plume is thought to have originated from releases at or from the sewer service line at a former dry cleaning service site and a former auto service station located on Gaffney Road between Noble and Turner Streets [37]. The eastern plume may also have contributed to the western plume through wood stave sewer lines [65]. Investigative, remedial, and monitoring work has occurred at Gaffney West since 1997 [36]. Three sources of PCE-contaminated soil were identified beneath and adjacent to the former Launderette building at the location of MW-9 between 1.5 to 3.7 m (5 to 12 ft) below ground surface [66], which was later occupied by a bookstore (on Gaffney Road, west of the four-plex). During a 2004 source characterization study, a wood stave secondary sanitary sewer line for the bookstore was breached, and 6.9 m^3 (9 cubic yards) of PCE-affected soil were removed. The western groundwater plume extends approximately 914 m (3,000 ft) from the Airport Way/Cushman Street intersection in a northwest direction toward the Chena River (Figure S1). VI assessments were completed at several buildings overlying the western plume (Table S2). A long-term study of the bookstore is reported in Barnes and McRae [25].

A soil vapor extraction/subslab depressurization system was installed in 2010 to mitigate the VI in the bookstore building. The system operated intermittently until 2018, when it was converted to a subslab depressurization system, only with a much smaller blower. Soil gas data showing concentrations of PCE up to 7,800 $\mu g/m^3$ in external soil gas in 2018 show that there is still a strong residual source present, which is likely to continue to contribute to the western plume [38].

A church also overlies the western plume and was historically sampled for vapor intrusion (Figure S1, Table S2). The church had a maximum subslab PCE concentration of 1,700 $\mu g/m^3$ and a maximum indoor air concentration of 0.68 $\mu g/m^3$ in the single round of previous sampling in 2007. Groundwater at the

aforementioned location is shallow, with depth to water at around 4.6 m (15 ft) below ground surface [36].

Building and HVAC characteristics

The buildings studied were selected to be of a larger, commercial scale because high-temporal resolution studies up to the present have primarily focused on residential-scale structures. Six commercial building structures between 149 and 6500 m^2 (1,600 and 70,000 ft^2 were selected for this study (Table S2). These structures include four buildings over the eastern plume and two buildings over the western plume. Building features and characteristics were recorded during site visits and walk-through inspections and recorded on survey forms similar to those recommended in the EPA VI Guidance [48]. The survey forms detail or confirm the layout, construction (e.g., slab-on-grade, crawl spaces), potential VOC sources (e.g., cleaning products, VOC sinks such as carpets, furniture, draperies), and operating processes (type of heating or cooling system, etc.) of the units that may influence contaminant entry. The spacing of relevant interior features was also documented, including the presence or absence of significant floor or basement wall cracks. Significant changes in the routine position or settings of interior and exterior doors, windows, and heating, ventilation, and air conditioning (HVAC) systems were documented on data collection forms and summarized in an event log. Building characteristics of each study building are individually described as follows. Note that during the study period, the building was operated as realistically as possible, with the operations controlled by the regular business or residential occupants.

A section of the building housing a not-for-profit was monitored during this study. It is a 369 m^2 (3,975 ft^2), single-story commercial structure that is part of the four-plex and is primarily used for food services. The structure was constructed in 1950 with cinder blocks and consists of a conference room, a kitchen, a dining room, a storage area, and bathrooms. The primary heating system for this building is through hot

air circulated via ducts and generated by two boiler systems that heat water to heat air. The building is cooled via central air and has other ventilation systems such as a kitchen hood fan and bathroom fans. Based on the building survey, a single HVAC zone was determined for this structure. HVAC zones (thermal zones) are spaces controlled with one thermostat typically served by a single air handling unit. VOC and radon sampling devices were deployed at several locations to characterize the indoor air and subslab. A total of two passive indoor air VOC samplers (Radiello®), two subslab VOC sample ports, and one radon sampler (RadonEye®) were deployed. One differential pressure sensor measuring subslab to indoor air pressure and two HOBO® temperature sensors collecting indoor and outdoor temperatures were also installed at this building.

A commercial tailor and tuxedo rental business, also located in the four-plex, resides at the second building in this study. This 454 m^2 (4,890 ft^2), single-story structure includes a storeroom, a tuxedo storage room, a central closet, an office, several bathrooms, and an apartment with a bathroom and a kitchen to the south of the building. The building is primarily heated through hot water baseboards generated by a boiler system with additional space heaters used in storage rooms. Although the building has access to central air cooling, it is not used. Additional ventilation systems include fans in storage rooms for in-room heating. VOC and radon sampling devices deployed at this building include two passive indoor air VOC samplers (Radiello®), three subslab VOC sample ports, and two radon samplers (RadonEye®). In addition, two differential pressure sensors, one measuring subslab to indoor air pressure and temperature and the other measuring indoor and outdoor air pressure, were deployed, along with an indoor air temperature device. Two groundwater monitoring wells, MW-33S and MW-31, located north and south of this building, respectively, were sampled every quarter.

Another structure included in this study is a 149 m^2 (1,600 ft^2), two-story residential-style office building with a basement and an attached garage on the east side of the building. The building is primarily used as an office space with several office rooms, a bathroom, a kitchen, and a break room on the first floor

and more office and storage rooms on the second floor. The concrete-floored basement accessed through the former attached garage is primarily used as a storage space and includes a closet and a boiler. The boiler provides hot water to radiators at baseboards to heat the building. Additional ventilation systems include fans in the bathrooms. No working cooling systems were documented. VOC and radon sampling devices were deployed at several locations in the basement and first floor to characterize the indoor air and subslab. The basement had one passive indoor air VOC sampler (Radiello®), two subslab VOC sample ports, and one radon sampler (RadonEye®) deployed. One passive indoor air VOC sampler (Radiello®) and one radon sampler (RadonEye®) were also deployed in the kitchen on the first floor. Two differential pressure sensors, one measuring subslab to indoor air pressure and temperature and the other measuring indoor and outdoor air pressure, were deployed in the basement and the garage, respectively. An indoor air temperature device (HOBO®) was also deployed in the kitchen on the first floor.

A government building is another structure monitored by this study. It is a two-story, approximately 650 m^2 (70,000 ft^2) building with a basement that is partially below grade. The building has several offices on the first and second floors and a gymnasium, a teen center, storage/file rooms, and a maintenance room, with the main air unit in the basement. The building is primarily heated through radiators receiving steam from the district steam plant. Building cooling is achieved through central air in some areas, with most offices having individual air conditioning units. Ventilation is achieved through large vents in most portions of the building (i.e., overhead or wall vents). VOC and radon sampling devices were deployed in the basement and first floor of the building for characterizing indoor air and subslab.

Another structure within this study is a two-story, 156 m^2 (1,680 ft^2) building primarily used as real estate office space on its first and second floors. This building is primarily heated through hot water radiators supplied by an on-site boiler and cooled using central air on the first floor with individual air

conditioning units used on the second floor. Additional ventilation systems include several bathroom fans on both floors. One passive indoor air VOC sampler (Radiello®) and one radon sampler (RadonEye®) are on the first and second floors. Two subslab ports that sampled VOCs and radon were also deployed on the first floor. Two differential pressure sensors were also installed on the first floor of this building. One sensor was installed to measure the differential pressure between the first and second floors, and the other sensor was installed in the boiler room to measure subslab to indoor air pressure. However, starting on January 14, 2021, a pressure sensor was installed to compare the pressure from inside the building in an adjacent, typically open room with the pressure outside, rather than the mechanical room, which is much warmer than the rest of the building and typically closed.

The Church is a 557 m^2 (6,000 ft^2), two-story building with a basement. Entry to the building is through a first-floor split-level with stairs leading to the basement and second floor. The presence of a basement sump and significant cracks were noted in the basement slab at the time of the initial survey. The building is primarily heated through hot water radiators supplied from the city's central hot water system. No known cooling systems were noted in the building, but bathroom vents with outside air intake were documented. Two passive indoor air VOC samplers (Radiello®) and one radon sampler (RadonEye®) were deployed in the basement, along with three subslab ports. Three differential pressure sensors were also deployed in the basement to measure subslab to indoor air, indoor to outdoor air, and basement to second floor differential pressures. An outside ambient air sampler was also deployed at the south end, away from the building. The high-ceilinged second floor had one passive indoor air VOC sampler (Radiello®) and one radon sampler (RadonEye®), along with an indoor air temperature sensor.

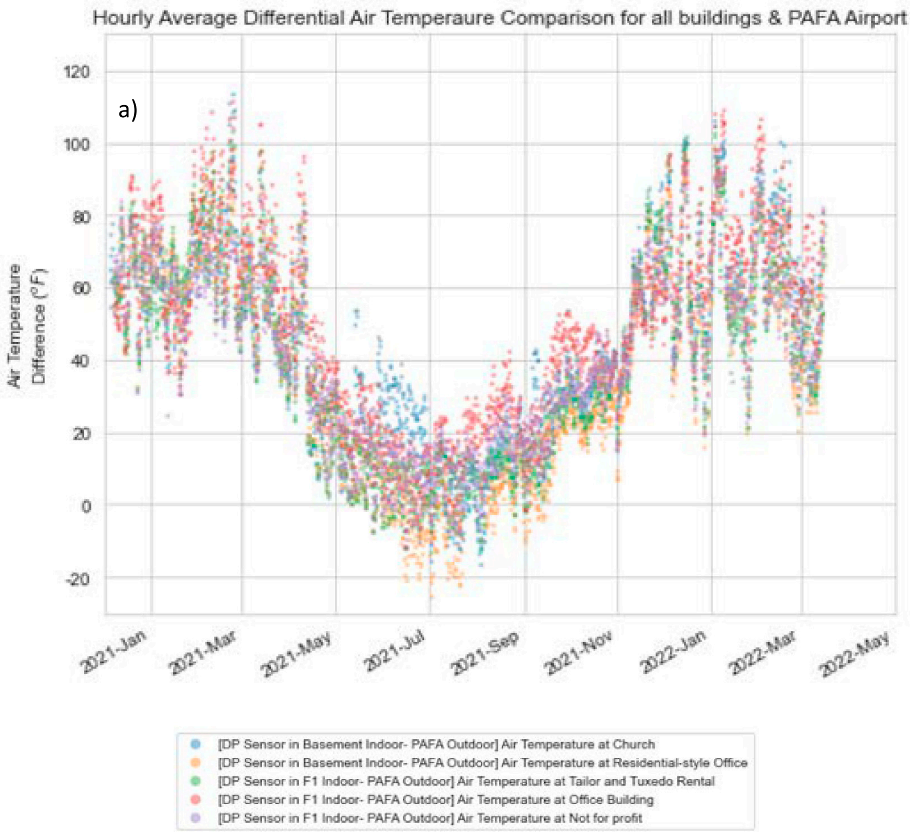
Hourly Average Differential Air Temperaure Comparison for all buildings & PAFA Airport
a)
Air Temperature Difference (°F)
120
100
80
60
40
20
0
-20
2021-Jan
2021-Mar
2021-May
2021-Jul
2021-Sep
2021-Nov
2022-Jan
2022-Mar
2022-May
[DP Sensor in Basement Indoor- PAFA Outdoor] Air Temperature at Church
[DP Sensor in Basement Indoor- PAFA Outdoor] Air Temperature at Residential-style Office
[DP Sensor in F1 Indoor- PAFA Outdoor] Air Temperature at Tailor and Tuxedo Rental
[DP Sensor in F1 Indoor- PAFA Outdoor] Air Temperature at Office Building
[DP Sensor in F1 Indoor- PAFA Outdoor] Air Temperature at Not for profit

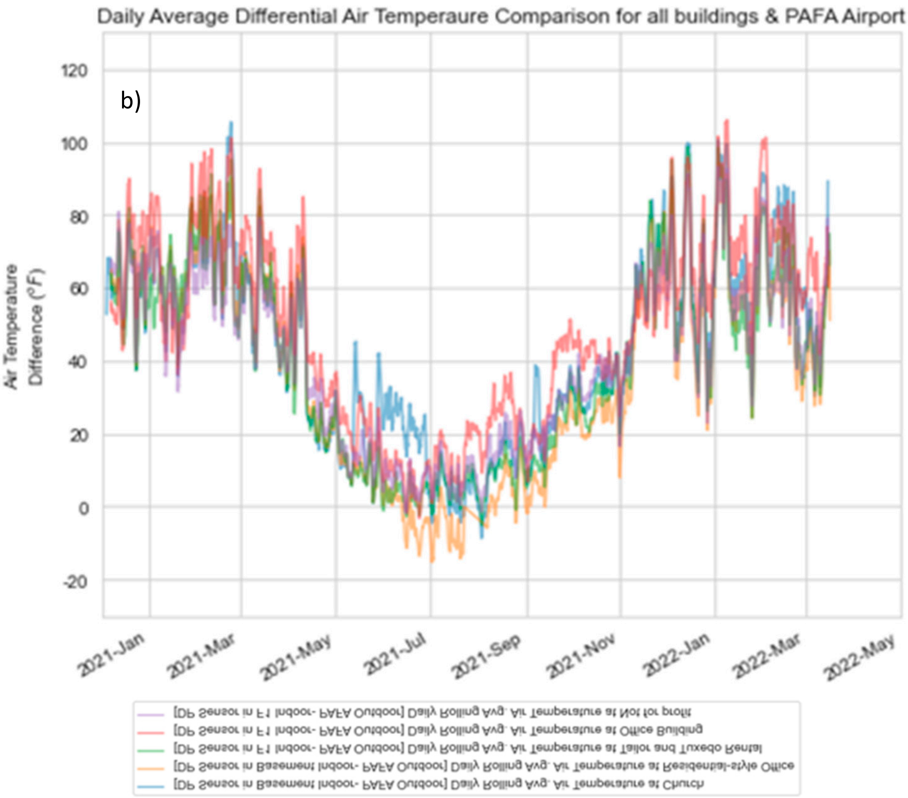
Daily Average Differential Air Temperaure Comparison for all buildings & PAFA Airport
b)
Air Temperature Difference (°F)
120
100
80
60
40
20
0
-20
2021-Jan
2021-Mar
2021-May
2021-Jul
2021-Sep
2021-Nov
2022-Jan
2022-Mar
2022-May
[DP Sensor in F1 Indoor- PAFA Outdoor] Daily Rolling Avg. Air Temperature at Not for profit
[DP Sensor in F1 Indoor- PAFA Outdoor] Daily Rolling Avg. Air Temperature at Office Building
[DP Sensor in F1 Indoor- PAFA Outdoor] Daily Rolling Avg. Air Temperature at Tailor and Tuxedo Rental
[DP Sensor in Basement Indoor- PAFA Outdoor] Daily Rolling Avg. Air Temperature at Residential-style Office
[DP Sensor in Basement Indoor- PAFA Outdoor] Daily Rolling Avg. Air Temperature at Church

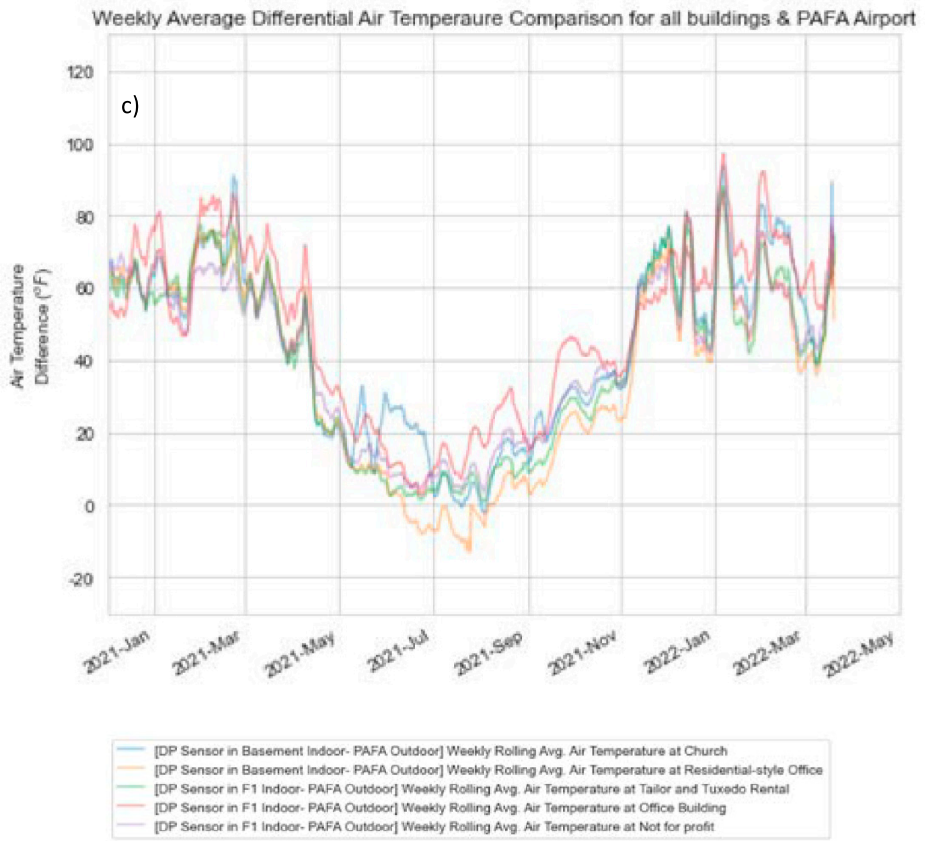

Figure S5. Differential temperatures, all buildings (a) every 6 hours, (b) daily average, and (c) weekly average.

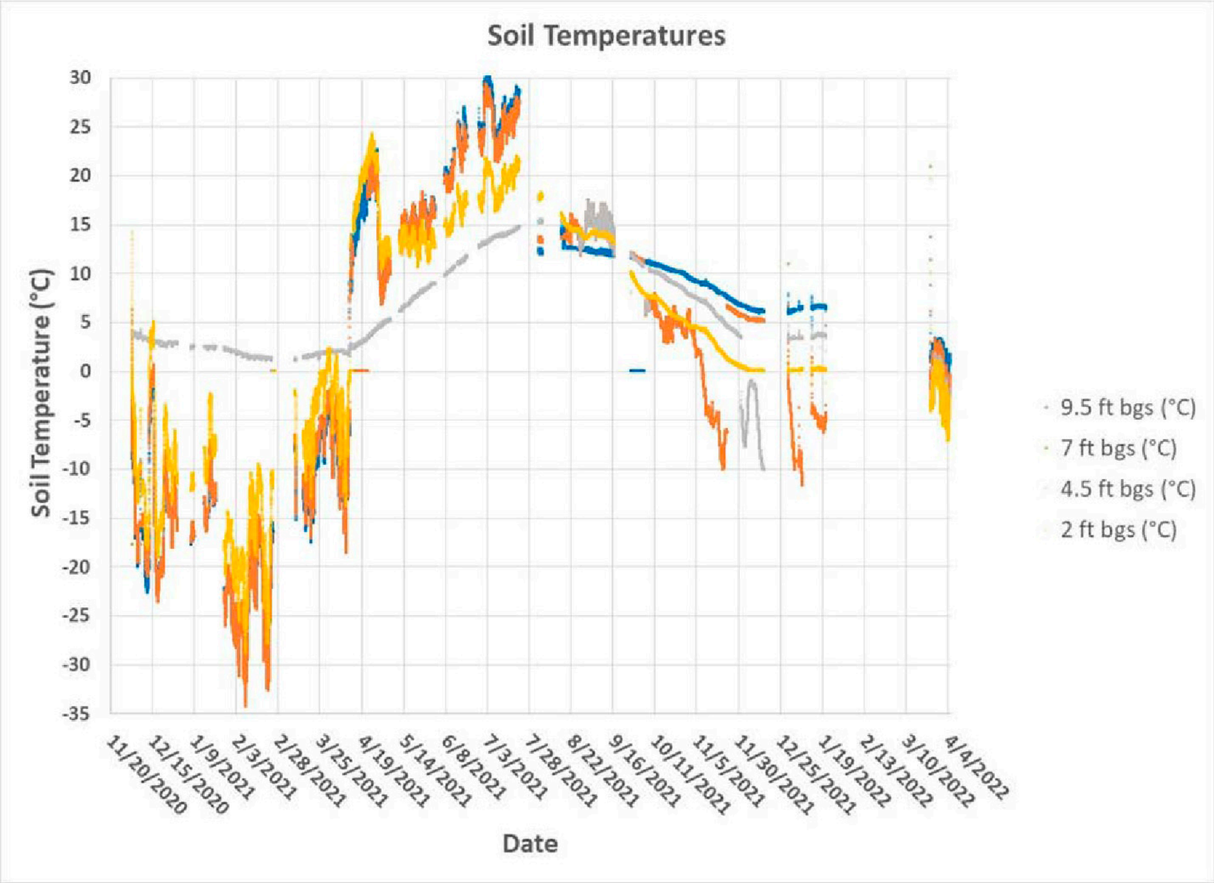

Figure S6. Soil temperatures

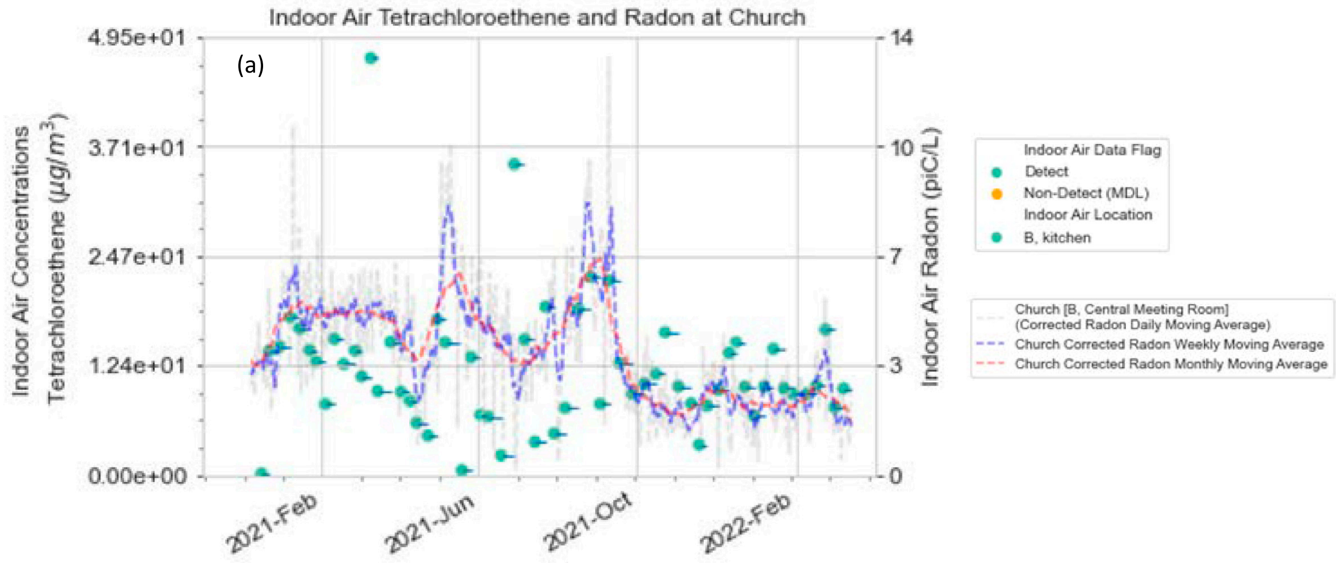

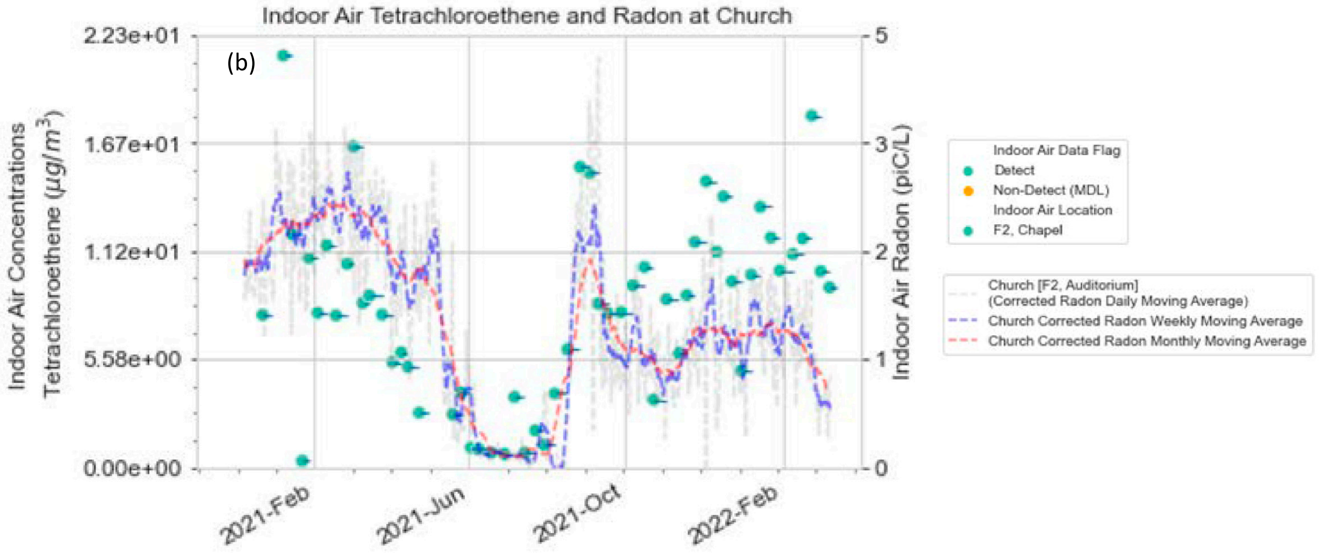

Indoor Air Tetrachloroethene and Radon at Church
(b)
Indoor Air Concentrations Tetrachloroethene ($\mu g/m^3$)
Indoor Air Radon (piC/L)
2.23e+01
1.67e+01
1.12e+01
5.58e+00
0.00e+00
5
3
2
1
0
2021-Feb
2021-Jun
2021-Oct
2022-Feb
Indoor Air Data Flag
Detect
Non-Detect (MDL)
Indoor Air Location
F2, Chapel
Church [F2, Auditorium]
(Corrected Radon Daily Moving Average)
Church Corrected Radon Weekly Moving Average
Church Corrected Radon Monthly Moving Average

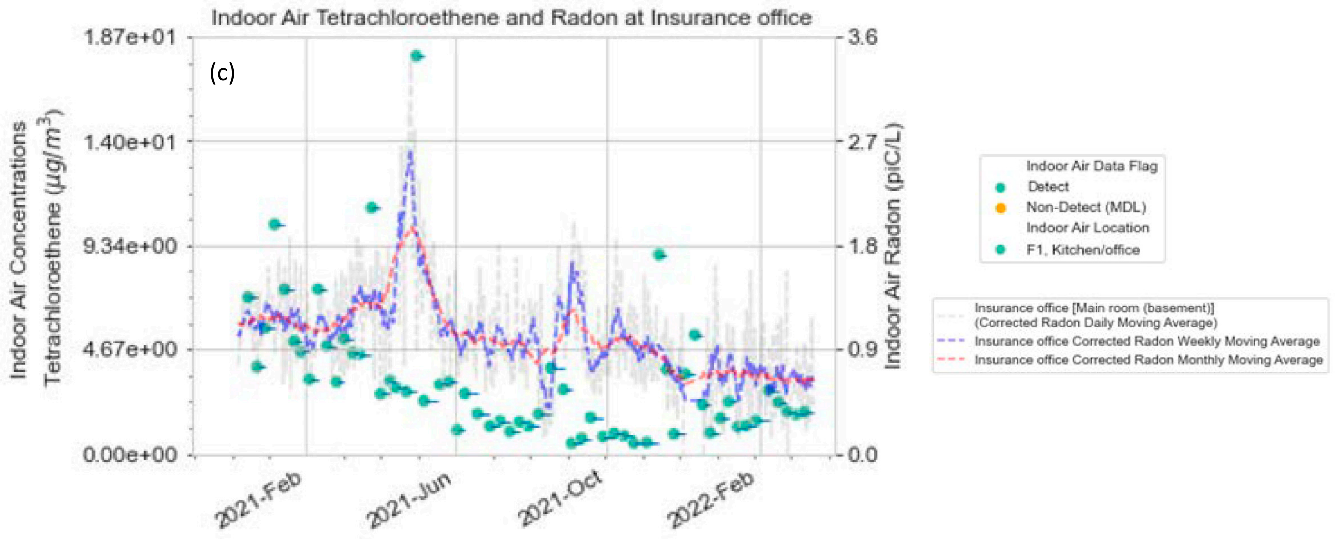

Indoor Air Tetrachloroethene and Radon at Insurance office
(c)
Indoor Air Concentrations Tetrachloroethene ($\mu g/m^3$)
Indoor Air Radon (piC/L)
1.87e+01
1.40e+01
9.34e+00
4.67e+00
0.00e+00
3.6
2.7
1.8
0.9
0.0
2021-Feb
2021-Jun
2021-Oct
2022-Feb
Indoor Air Data Flag
Detect
Non-Detect (MDL)
Indoor Air Location
F1, Kitchen/office
Insurance office [Main room (basement)]
(Corrected Radon Daily Moving Average)
Insurance office Corrected Radon Weekly Moving Average
Insurance office Corrected Radon Monthly Moving Average

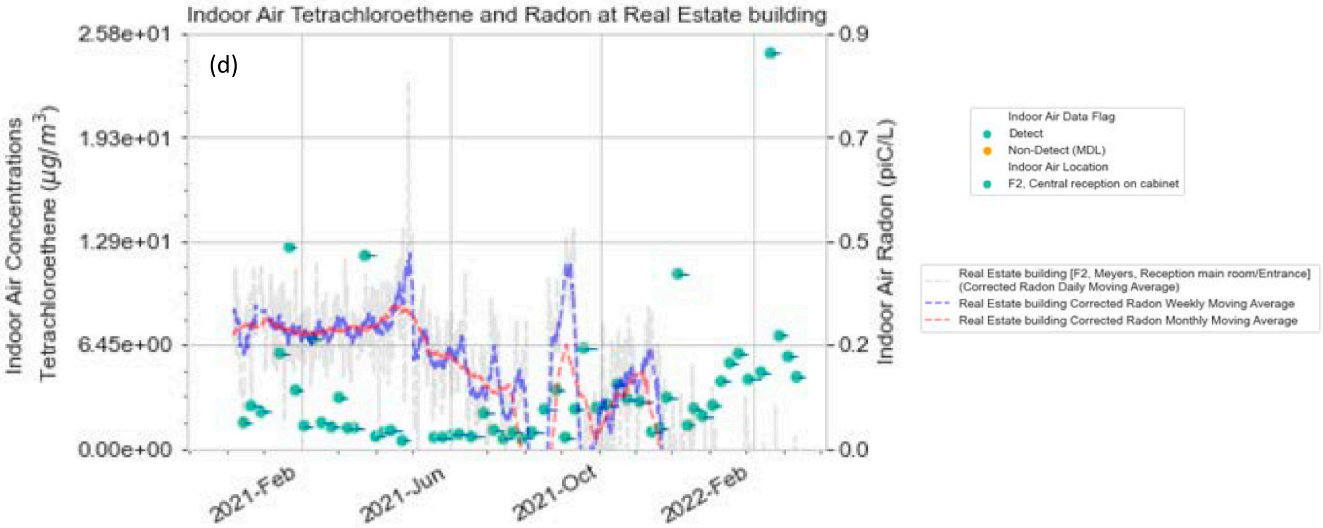

Indoor Air Tetrachloroethene and Radon at Real Estate building
(d)
2.58e+01
1.93e+01
1.29e+01
6.45e+00
0.00e+00
0.9
0.7
0.5
0.2
0.0
Indoor Air Concentrations Tetrachloroethene ($\mu g/m^3$)
Indoor Air Radon (piC/L)
2021-Feb
2021-Jun
2021-Oct
2022-Feb
Indoor Air Data Flag
Detect
Non-Detect (MDL)
Indoor Air Location
F2, Central reception on cabinet
Real Estate building [F2, Meyers, Reception main room/Entrance] (Corrected Radon Daily Moving Average)
Real Estate building Corrected Radon Weekly Moving Average
Real Estate building Corrected Radon Monthly Moving Average

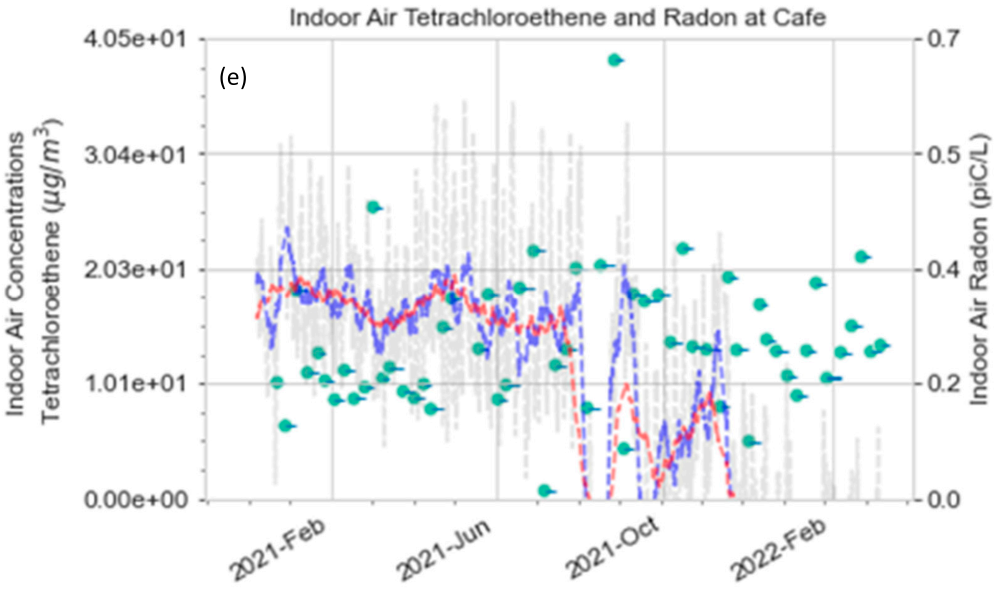

Indoor Air Tetrachloroethene and Radon at Cafe
(e)
4.05e+01
3.04e+01
2.03e+01
1.01e+01
0.00e+00
0.7
0.5
0.4
0.2
0.0
Indoor Air Concentrations Tetrachloroethene ($\mu g/m^3$)
Indoor Air Radon (piC/L)
2021-Feb
2021-Jun
2021-Oct
2022-Feb

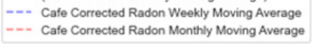

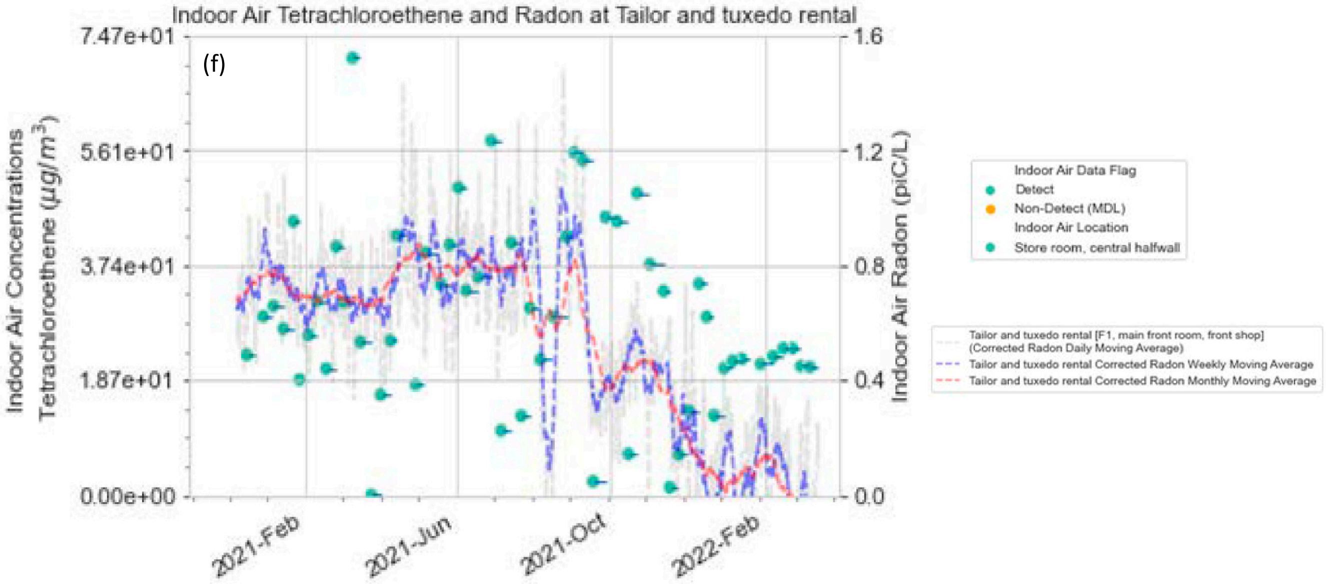

Figure S7. Stacked PCE and radon plots, by floor, by building: (a) Church, Central Meeting Room (Basement); (b) Church, Auditorium (2nd floor); (c) Insurance Office (basement); (d) Real Estate Office (2nd floor); (e) Not-for-Profit; (f) Tailor and Tuxedo Rental.

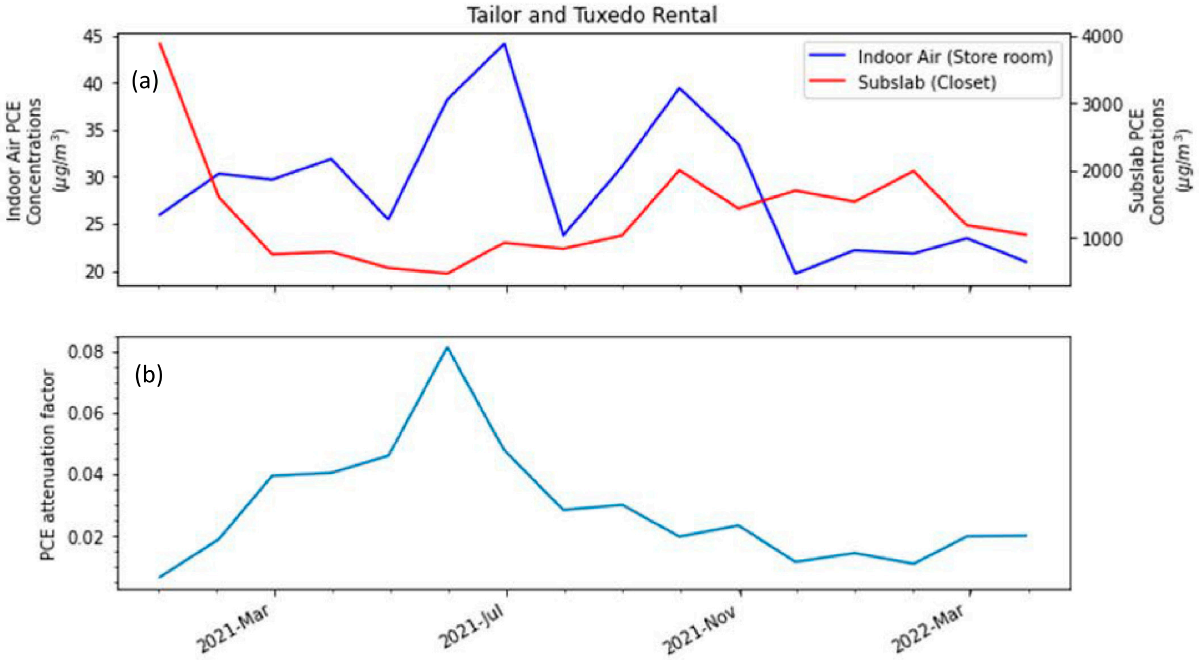

Figure S8. Tailor and Tuxedo Rental building, (a) indoor air concentrations at the store room and subslab PCE concentrations in the closet, averaged by month; (b) PCE attenuation factor from subslab to indoor air calculated on a month by month basis.

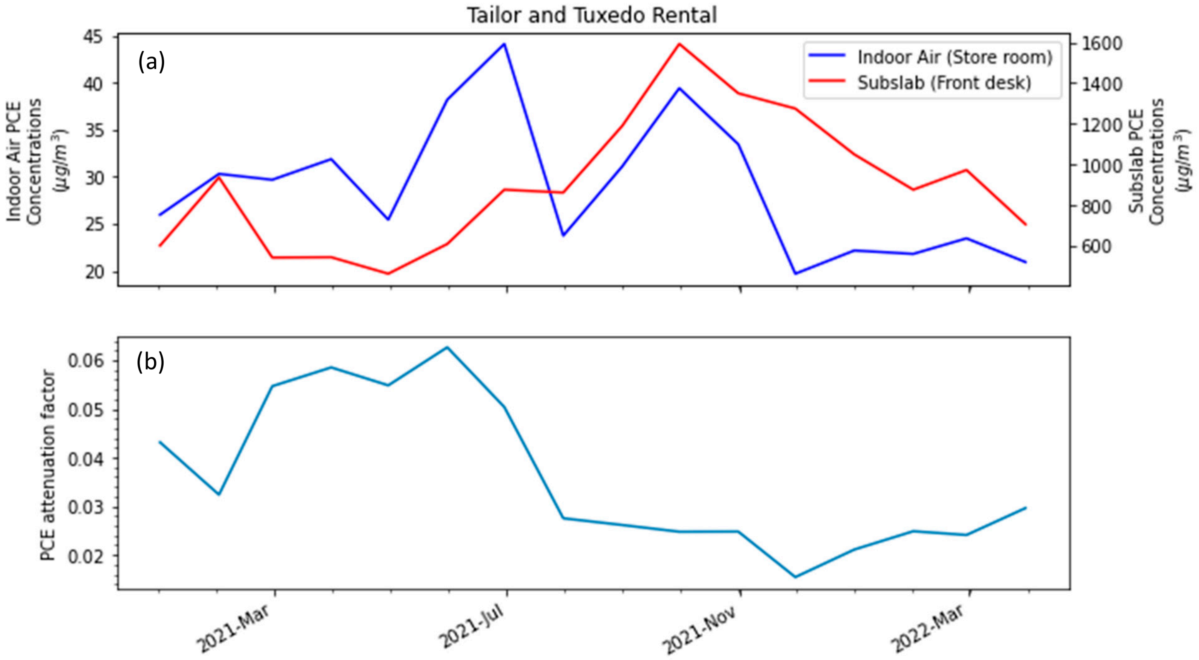

Figure S9. Tailor and tuxedo rental building, (a) indoor air concentrations at the store room and subslab PCE concentrations at the front desk, averaged by month; (b) PCE attenuation factor from subslab to indoor air calculated on a month by month basis.

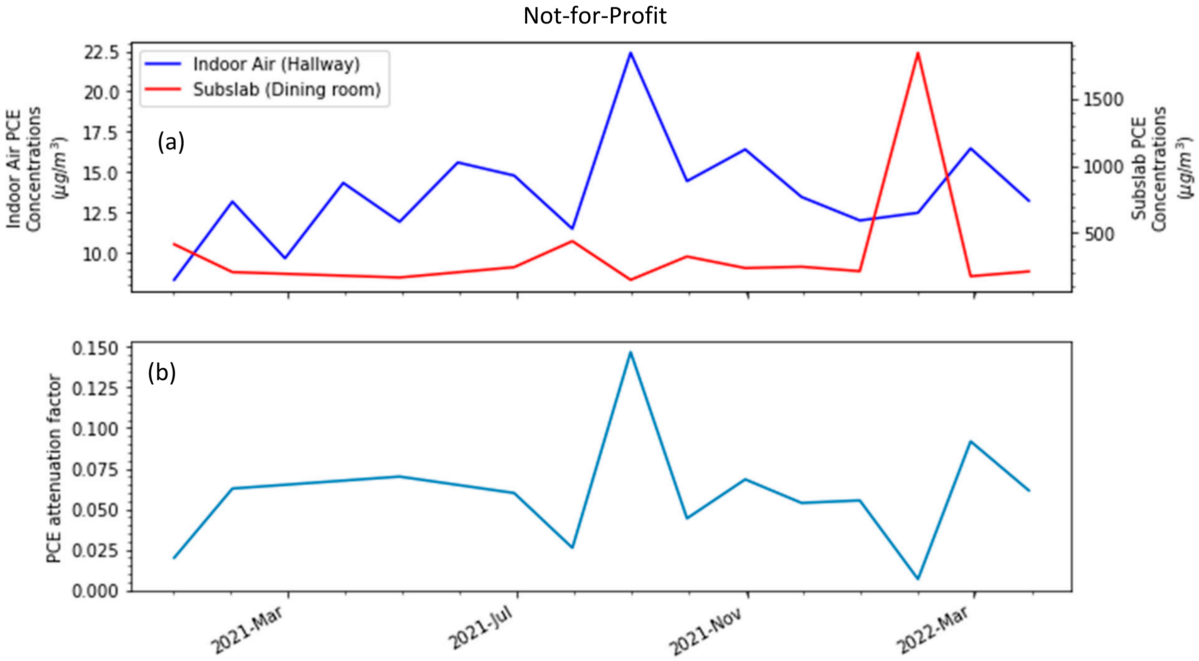

Figure S10. Not for profit building, (a) indoor air concentrations at the hallway and subslab PCE concentrations at the dining room, averaged by month; (b) PCE attenuation factor from subslab to indoor air calculated on a month by month basis.

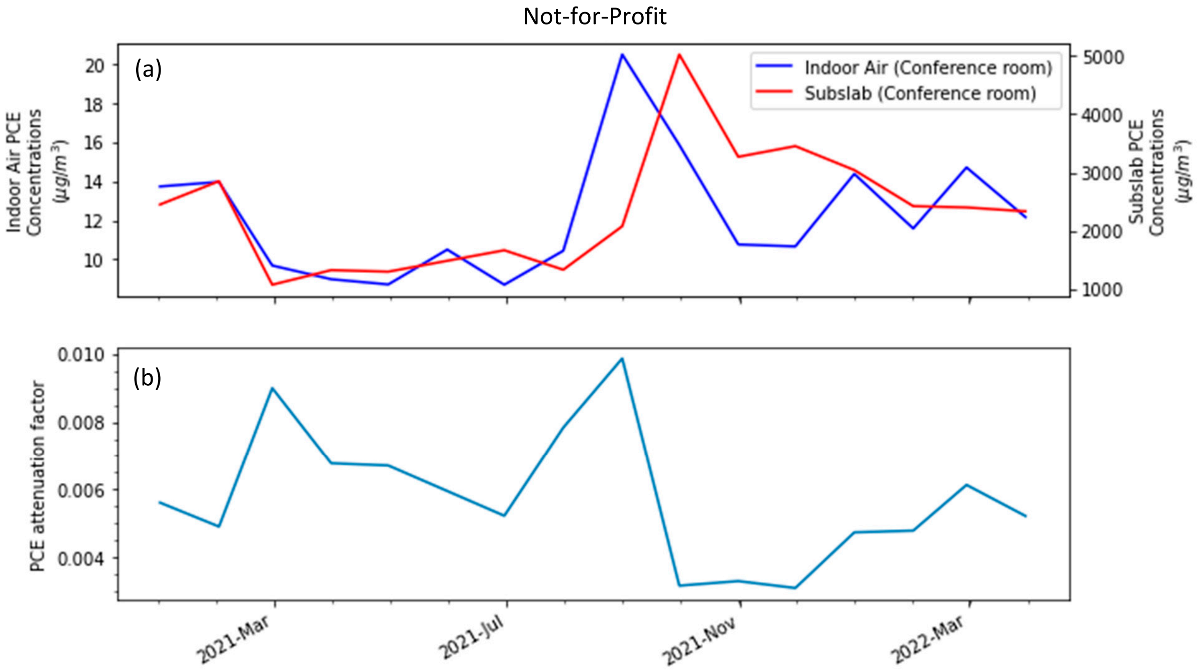

Figure S11. Not-for-Profit building, (a) indoor air and subslab PCE concentrations in the conference room, averaged by month; (b) PCE attenuation factor from subslab to indoor air calculated on a month by month basis.

References

25. Barnes, D.L.; McRae, M.F. The predictable influence of soil temperature and barometric pressure changes on vapor intrusion. Atmos. Environ. 2017, 150, 15–23. https://doi.org/10.1016/j.atmosenv.2016.11.018.

36. Oasis Environmental. Fiscal Year 2011 Vapor Intrusion and Groundwater Monitoring Report; Gaffney Road East Area-Wide In-vestigation: Fairbanks, AK, USA, 2011. Available online: https://dec.alaska.gov/Applications/SPAR/PublicMVC/CSP/Download?documentID=58363&fileName=2919_2011%20West%20VI%20and%20GW%20Report%20Final.pdf (accessed on 5 March 2025).

37. Ecology and Environment, Inc. Site Inspection, Coin King Laundromat; Gaffney Road East Area-Wide Investigation: Fairbanks, Alaska, 2014. Available online: https://dec.alaska.gov/Applications/SPAR/PublicMVC/CSP/SiteReport/25573 (accessed on 5 March 2025).

38. Ahtna Environmental. State Fiscal Year 2017 Groundwater Monitoring, Soil Gas Monitoring, Well Inventory, Remedial System Evaluation, and Mitigation System Operations and Maintenance Report; State of Alaska Department of Environmental Conserva-tion: Fairbanks, AK, USA, 2018.

48. U.S. Environmental Protection Agency. Compendium Method TO-17, Determination of Volatile Organic Compounds in Am-bient Air Using Active Sampling onto Sorbent Tubes, Washington,

DC, 1999. USEPA Method TO-17. Available online: https://www.epa.gov/sites/default/files/2019-11/documents/to-17r.pdf (accessed on 5 March 2025).
59. Oasis Environmental. Additional Source Characterization and Long Term Monitoring; Gaffney Road Area: Fairbanks, AK, USA, 2009. Available online: https://dec.alaska.gov/Applications/SPAR/PublicMVC/CSP/Download?documentID=58372&fileName=25573_2009_%20Final%20Report%20Addtl%20Characterization%20and%20LTM.pdf (accessed on 5 March 2025).
62. Ecology and Environment, Inc. Site inspection: Coin King Laundromat; Technical Direction Document Number: 13-8-0036; United States Environmental Protection Agency: Fairbanks, AK, USA, 2014.
63. Oasis Environmental. Additional Site Characterization and Long-Term Monitoring Gaffney Road Area; Oasis Environmental: Fair-banks, AK, USA, 2009. Available online: https://dec.alaska.gov/Applications/SPAR/PublicMVC/CSP/Download?documentID=58371&fileName=2919_2009_%20Final%20Report%20Addtl%20Characterization%20and%20LTM.pdf (accessed on 5 March 2025).
64. ERM Alaska. State Fiscal Year 2013, Operations and Maintenance of Remediation System and Additional Characterization Report; Gaffney Road West: Fairbanks, AK, USA, 2013. Available online: https://dec.alaska.gov/applications/spar/publicmvc/csp/sitereport/2919 (accessed on 5 March 2025).
65. Ecology & Environment. Gaffney Road Area-Wide Phase II Groundwater Investigation, Fairbanks, Alaska; Final Report; ADEC: Anchorage, AK, USA, 1999.
66. Howe, J.W. The Houses We Live in: An Identification Guide to the History and Style of American Domestic Architecture; Thunder Bay Press: San Diego, CA, USA, 2002.
67. Fairbanks North Star Borough. Property Tax Database. Available online: https://propertysearch.fnsb.gov/ (accessed on 7 April 2023).
68. Weller, A. (Ahtna Engineering Services, Fairbanks, AK, USA); Chris Lutes (Jacobs Engineering, Cary, NC, USA). Personal Communication, 2020.
